# FADD regulates NF-κB activation and promotes ubiquitination of cFLIP_L_ to induce apoptosis

**DOI:** 10.1038/srep22787

**Published:** 2016-03-14

**Authors:** Kishu Ranjan, Chandramani Pathak

**Affiliations:** 1 Department of Cell Biology, School of Biological Sciences and Biotechnology, Indian Institute of Advanced Research, Koba Institutional Area, Gandhinagar-382007, Gujarat, India

## Abstract

Tumor Necrosis Factor-α canonically induces the activation of NF-κB and associated gene product cellular FLICE-like inhibitory protein (cFLIP_L_) to promote cell survival. Previously, we demonstrated that ectopic expression of the Fas associated death domain (FADD) diminishes the expression of cFLIP_L_ and transduces caspases-8 mediated apoptosis, independent of FasL stimulation in HEK 293T cells. However, the underlying molecular mechanism of FADD mediated ablation of cFLIP and NF-κB signaling to determining the fate of cell death or survival remains elusive. Here, we explored a novel molecular mechanism of FADD mediated apoptotic cell death that was directed by ubiquitination of cFLIP_L_ and inhibition of NF-κB activation, independent of TNF-α stimulation. We found that induced expression of FADD firmly interacts with procaspase-8 and precludes cFLIP_L_ to from the death inducing signaling complex (DISC). In addition, FADD negatively regulates cellular inhibitor of apoptosis protein 2 (cIAP2) and Bcl-2. Furthermore, FADD restrains cIAP2 expression and interacts with RIP1 and procaspase-8 to accomplish apoptotic cell death signaling. Interestingly, FADD was also found to promote JNK1 mediated activation of E3 ubiquitin ligase ITCH to degrade cFLIP_L_ that may lead to commencement of apoptosis. Thus, FADD is an important regulator for determining the fate of cell death or survival.

Fas associated death domain (FADD) is a pivotal signaling component of death receptor (DR) mediated apoptosis. DRs such as Fas (CD95/Apo) and tumor necrosis factor receptor 1 (TNFR1) (p55/CD120a), belongs to the TNF receptor super family that contain cytoplasmic death domain (DD) to execute downstream signal transduction^1^. Upon binding of ligand to the cell surface receptors, the DD of cell surface receptor homophilically interacts with the DD of FADD and induces oligomerization of DED (death effector domain) of FADD with apical caspases such as, procaspase 8/10 to form a death-inducing signaling complex (DISC)^2^. In the downstream, DISC facilitates processing and catalytic activation of caspases-8/10 to transduces downstream signaling of apoptosis^3^. However, the catalytic activation of caspase-8/10 has been negatively regulated by the anti-apoptotic protein Cellular Flice like inhibitory protein (cFLIP) to abrogate apoptotic instigation^4^. Although FADD is a multifunctional protein and its Fas ligand mediated proapoptotic function has been well studied^5^,^6^. However, the cellular dynamics of FADD and cFLIP in the regulation of cell death and survival by TNFR signaling remains elusive. TNF receptor (TNFR) signaling elicits both non-apoptotic and apoptotic response by the formation of two sequential complexes depending upon the stimulation of the TNF-α. The components of complex I constituted with TRADD, TRAF2, cIAPs and RIP1 activates NF-κB signaling for promoting cell survival. However, the subsequent dissociation of RIP1 from complex I and association with FADD and procaspase-8 initiates formation of pro-apoptotic complex II that substantiates apoptotic cell death^7^. Although, TNF-α augments the activation of transcription factor NF-κB in tumor cells and promotes cell proliferation by impeding apoptosis^8^. The TNF-α-induced NF-κB activation confers upregulation of several anti-apoptotic genes such as *Bcl-xL*, *A1/Bfl-1*, (*c-IAP)1/2*, X-chromosome-linked IAP (*XIAP*; also known as *hILP*) and *cFLIP*^9^,^10^,^11^. Surprisingly, upregulation of cFLIP_L_ also provides strength to TNFR1 signaling for stability of complex I formation and constitutive NF-κB activation along with cell proliferation and survival^12^,^13^. Indeed, the constitutive activation of NF-κB suppresses immune surveillance in both adaptive and innate response and supports tumorigenesis, cancer chemoresistance and chronic inflammation^14^,^15^,^16^. Moreover, the growth and cell survival connecting signaling pathways such as MAPK/ERK and Akt are also associated with transcriptional regulation of NF- κB and cFLIP_L_^12^. Considering that NF-κB and cFLIP importantly serve as the molecular switches for pro-survival signaling.

In fact, TNF-α mediated NF-κB and JNK signaling pathways are corroborated with cell death and survival^17^. Previously it has been shown that, prolong stimulation of TNF-α arbitrates the phosphorylation and activation of Jun kinase (JNK) to promote reactive oxygen species (ROS) and cell death^9^,^18^,^19^. Interestingly, the relevance of the JNK activation and apoptotic cell death are also controlled by NF-κB signaling^20^. However, the cellular mechanism of programmed cell death and survival regulated by NF-κB and JNK1 in connection with FADD remains to be elucidated. A recent report suggests that TNF-α can induce RIP1 dependent programmed necrosis during an inactive state of caspases-8^21^. Interestingly, FADD negatively regulates RIP1/3 dependent necroptosis^22^,^23^. Moreover, the cytosolic availability of FADD and deubiquitination of RIP1 provides stability to complex II and negatively acts on the integrity of complex I^7^,^24^. Although, the stability of complex I provided by FLIP_L_ is largely affected by activation of JNK1 and E3 ubiquitin ligase ITCH that facilitates proteosomal degradation of cFLIP_L_^25^,^26^. Previously, we have shown that induced expression of FADD alleviates the expression of cFLIP_L_ and activates caspase-8 mediated apoptosis in HEK 293T cells^27^. The present study was aimed to delineate the molecular signaling mechanism acquired by FADD in regulation of two sequential complexes originating from a common axis of TNF receptors. We observed that induced expression of FADD regulates NF-κB activation and cIAP2 and cFLIP_L_ expression accompanied with JNK1 mediated ubiquitination of cFLIP_L_ to commence apoptotic cell death signaling.

## Results

### Sufficient availability of FADD ablates cFLIP_L_ binding at the DISC

**Figure 1:**
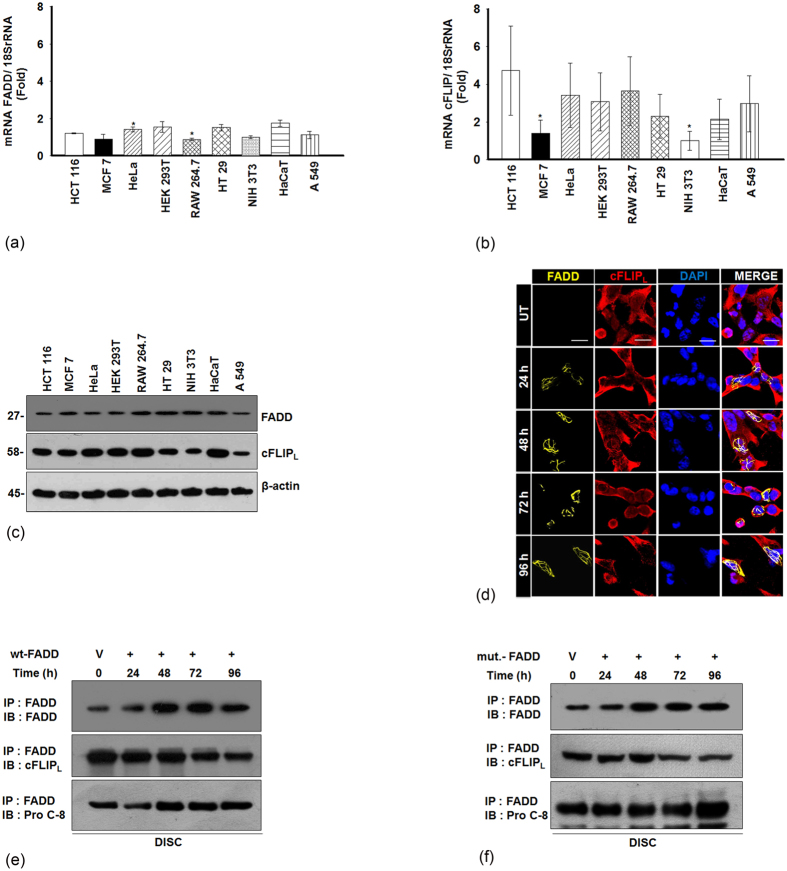
Expression of FADD and cFLIP_L_ in different cell lines and analysis of DISC assembly. Total mRNA was extracted from mentioned cell lines and RT-qPCR analysis, (**a**) mRNA expression of endogenous FADD and (**b**) mRNA expression of endogenous cFLIP_L_. The values were normalized by using the difference in critical thresholds (CT) between target gene and 18S rRNA (endogenous control). The expression of mRNA of the respective genes was compared with non-cancerous NIH 3T3 cells as a control using the values of 2^−ΔΔCT^. (**c**) The total cell lysate was extracted from the mentioned cell lines and the expression of endogenous FADD and cFLIP_L_ were monitored by Western blotting. The uncropped full-length blots are presented in supplementary Fig. S2. (**d**) Immunostaining of cFLIP_L_ in pEYFP-FADD overexpressed HEK 293T cells, untransfected (UT) HEK 293T cells were taken as control, scale bar- 5 μm. DISC analysis in, (**e**) The pcDNA-FADD plasmid and (**f)** The pcDNA-FADD-SLT4 (mut. FADD- mutation in the DED of FADD; **S16T, S18T and L20V**) plasmids, transfected to HEK 293T cells and expressed for 24–96 h, post incubation total cell lysate was extracted and subjected to co-immunoprecipition assay. The binding of FADD, caspase-8 and cFLIP_L_ at DISC was detected by western blotting. The vector (V) pcDNA 3.1vector transfected HEK 293T cells for 48 h (shown as 0 h time point) were taken as a control. The uncropped full-length blot of wild type FADD and procaspase-8 co-precipitated with mut. FADD are presented in supplementary Fig. S2. Error bars represent mean ± SD; *P ≤ 0.05, NIH 3T3 vs. cancer/ transformed cells (n ≥ 3, where n is the number of independent experiments), IP = immunoprecipitation; IB = immunoblotting.

A growing body of evidence demonstrates that elevated expression of cFLIP_L_ competitively excludes the interaction of initiator procaspase-8/10 with adaptor protein FADD at DISC to block death receptor signaling of apoptosis^28^,^29^,^30^. Indeed, our previous finding revealed that induced expression of FADD diminishes the expression of cFLIP_L_ and activates downstream cascade of caspases for execution of apoptosis in HEK 293T cells^27^. Although, previous reports suggest that low expression of FADD and elevated expression of cFLIP blocks apoptosis and promotes malignancy^2^,^31^. First, we examined here, the endogenous expression of FADD and cFLIP_L_ in different origins of cancer and transformed cells. We found an inadequate expression of FADD and elevated expression of cFLIP_L_ in cancer and transformed cells as compared to non-cancerous NIH 3T3 cells (Fig. 1a–c). Next, the subcellular localization of FADD was examined in HEK 293T cells by immunostaining. Notably, expression of endogenous FADD was only detected in the nuclear region, whereas transient expression of the FADD (post 48 h) was observed at the periphery of the nucleus and throughout the cytoplasmic space of cells (Fig. S1a). Next, we investigated the integrity of cFLIP_L_ during induced expression of FADD from 24–96 h by immunostaining. It was observed that the integrity of cFLIP_L_ was severely challenged when expression of FADD was progressed from 48 h onwards with concomitant progression of apoptotic cell death (Fig. 1d; Fig. S1b,c). Simultaneously, we analyzed the interaction of cFLIP_L_ and procaspase-8 with FADD at the DISC by co-immunoprecipitation assay. We observed that sufficient availability of FADD allows progressive binding of procaspase-8 at the DISC rather than the cFLIP_L_ (Fig. 1e). Further, this was confirmed by expressing a mutant of the FADD (FADD-SLT4; carrying mutation in the death effector domain^32^) in HEK 293T cells. We observed that mutations in DED of the FADD at Serine 16, Serine 19 and lysine 20 did not affect the binding of procaspase-8, but restrict the binding of cFLIP_L_ at the DISC (Fig 1f). Thus, FADD is an essential component for binding of cFLIP_L_ to regulate cell death or survival. Intrestingly, these results demonstrate that induced expression of FADD profoundly interacts with procaspase-8 and exclude cFLIP_L_ from the DISC to execute apoptotic cell death.

### FADD inhibits NF-κB activation with subsequent ablation of cFLIP_L_, independent of TNF-α stimulation

**Figure 2:**
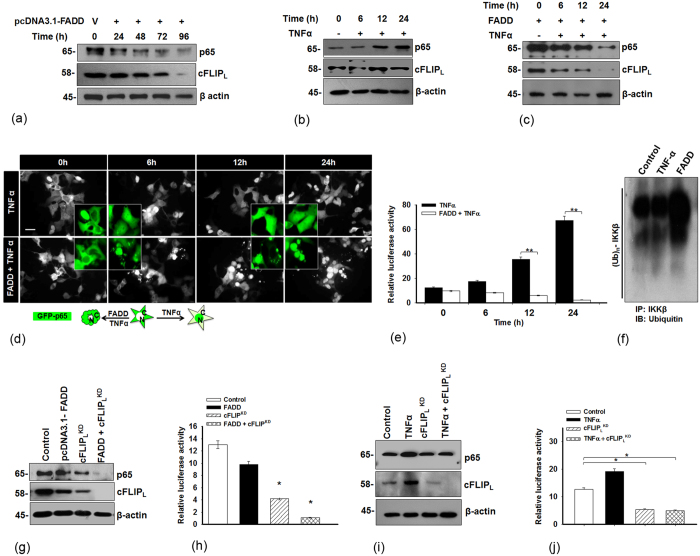
Induced expression of FADD inhibits NF-κB activation independent of TNF-α stimulation. (**a)** HEK 293T cells were transfected with pcDNA3.1-FADD and expressed for 24–96 h, control represents vector transfected cells. Expression of p65 and cFLIP_L_. (**b**) HEK 293T cells were exposed to TNF-α (10 ng/ml) for 6–24 h, control represents TNF-α untreated cells (shown as 0 h time point), Expression of p65 and cFLIP_L_ (**c**) The 48 h of pcDNA3.1-FADD transfected HEK 293T cells were subjected to TNF-α (10 ng/ml) treatment for 6–24 h. Control represents HEK 293T cells without TNF-α treatment and 48 h pcDNA-FADD transfected HEK 293T cells (shown as 0 h time point). Expression of p65 and cFLIP_L_, **(d)** GFP-p65 Cyto-nuclear translocation (scale bar-5 μm) and (**e**) NF-κB luciferase assay. (**f**) HEK 293T cells were subjected to TNF-α (10 ng/ml for 12 h) and FADD transfection (48 h), Control represents vector transfected cells without TNF-α, the ubiquitination of IKKβ was examined by co-immunoprecipitation followed by Western blotting. (**g**) HEK 293T cells transfected with pcDNA3.1-FADD (*lane 2*), siRNA directed against cFLIP_L_ (cFLIP_L_^KD^; *lane 3*) and pcDNA3.1-FADD with cFLIP_L_^KD^ (FADD + cFLIP_L_^KD^; *lane 4*), control represents vector and nontargeting siRNA transfected cells (*lane1*) for 48 h. Expression of p65 and cFLIP_L_ and (**h**) NF-κB luciferase assay. **(i)** HEK 293T cells were subjected to TNF-α (10 ng/ml) for 12 h (*lane 2*), cFLIP_L_^KD^ (*lane 3*) for 48 h and 12 h of TNF-α (10 ng/ml) primed followed by cFLIP_L_^KD^ (*lane 4*) incubated for 48 h. Control represents untreated and nontargeting siRNA transfected cells *(lane 1)*. Expression of p65 and cFLIP_L_ and **(j)** NF-κB luciferase assay. The uncropped full-length blots of Fig. (**a**,**c**,**g**) are presented in supplementary Fig. S5. Error bars represent mean ± SD, In (**e**), **P ≤ 0.001, TNF-α treatment in non-transfected vs FADD transfected cells (One way ANOVA followed by Student Newman-Keuls test, n = 4); In (**h**) *P ≤ 0.05, control vs FADD or cFLIP_L_^KD^ or FADD + cFLIP_L_^KD^, (student t-test, n = 4), In (**j**) *P ≤ 0.05, control vs TNF-α or cFLIP_L_^KD^ or TNF-α + cFLIP_L_^KD^, (student t-test, n = 4), where n is the number of independent experiments.

TNF-α triggers an intracellular cascade of signaling that plays an important role in determining the fate of cell death or survival^33^. Although, an activation of NF-κB signaling by TNF-α renders apoptosis by up regulating the anti-apoptotic genes such as *cIAPs, XIAPs and cFLIP* etc^9^. Moreover, the cFLIP is a known modulator of NF-κB activation and extrinsic signaling of apoptosis^11^,^34^. The above mentioned results showed that induced expression of FADD restricts binding of cFLIP_L_ at the DISC. Therefore, we were interested to examine the involvement of FADD in regulation of anti-apoptotic signaling of NF-κB in TNF-α stimulated cells. We found that, induced expression of FADD in HEK 293T cells downregulates the cytosolic expression of p65 and cFLIP_L_ as time progresses from 48 h onwards (Fig. 2a). Next, HEK 293T cells were exposed to TNF-α for 6–24 h and the activation of NF-κB and cFLIP_L_ were examined. As expected, expression of p65 was up regulated in response to TNF-α, in contrast, moderate changes were observed in the level of cFLIP_L_ (Fig. 2b). Surprisingly, exposure of TNF-α to 48 h of FADD expressed HEK 293T, MCF-7 and HCT 116 cells were not able to canonically protect the expression of p65 and cFLIP_L_ (Fig. 2c; Fig. S3a,c). Similarly the nuclear translocation of GFP-tagged p65 and NF-κB luciferase reporter assay in HEK 293T, MCF-7 and HCT 116 cells showed that FADD abolishes TNF-α induced NF-κB activation (Fig. 2d,e; Fig. S3b,d). In addition, we found that induced expression of FADD ubiquitinated and degraded IKKβ (regulator of p65 canonical inhibitor IκBα), that was protected in TNF-α treated and untreated cells (Fig. 2f). Further, the expression of cFLIP_L_ was knocked down ^(KD)^ by siRNA to monitor the expression of p65 and NF-κB Luciferase reporter activity in HEK 293T cells. We found that transient silencing of cFLIP_L_ negatively acts on the expression of p65 and NF-κB activity (cFLIP_L_^KD^; lane 3), and the effect was more radical upon cFLIP_L_ knockdown in FADD expressed HEK 293T cells (FADD + cFLIP_L_^KD^; lane 4) (Fig. 2g,h; Fig. S3e–g). Next, we were prompted to examine the stability of NF-κB and cFLIP by pre-exposure of TNF-α for 12 h followed by silencing of cFLIP_L_ using SiRNA in HEK293T cells. We found that pre-exposure of TNF-α was sufficient to raise the levels of p65 and cFLIP_L_ *(lane 2),* but failed to maintain the level upon challenging the expression of cFLIP_L_ (TNF-α + cFLIP_L_^KD^; lane 4) (Fig. 2i,j; Fig. S3h). Altogether, these results indicate that cFLIP_L_ acts as an essential component to strengthening NF-κB signaling, but FADD has the enormous potential to abrogate NF-κB activation and cFLIP_L_ expression independent of TNF-α.

### Mutation in the DED domain of FADD and cFLIP_L_ modulates NF-κB activation

**Figure 3:**
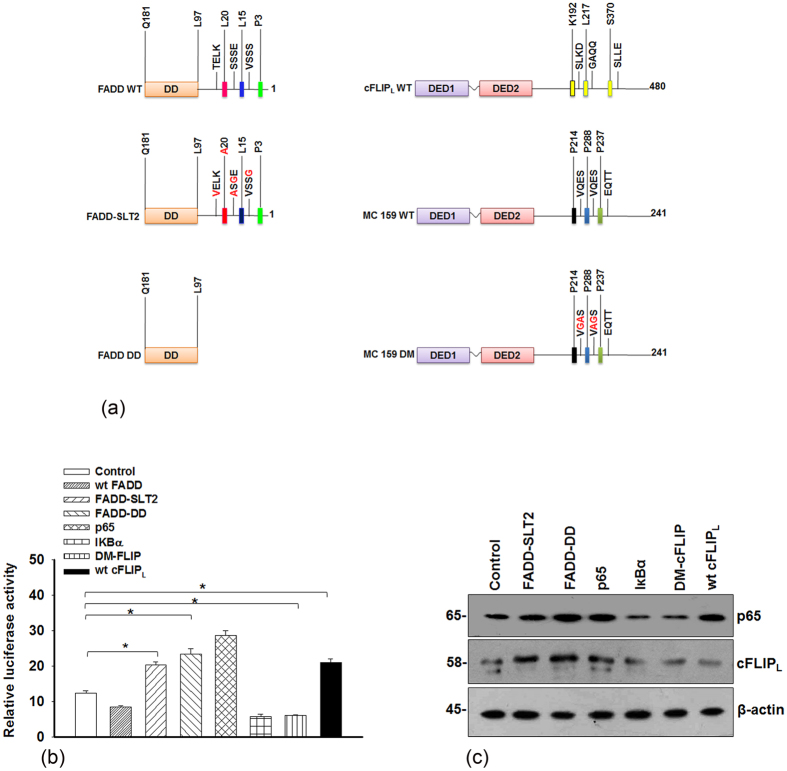
Mutation of specific amino acids in the FADD and cFLIP_L_ modulates NF-κB activity. (**a**) Schematic diagram of pcDNA -FADD wild type (WT), mutated FADD (FADD-SLT2; **S14G, S16A, S18G, L20A and T21V**), death domain (DD) of the FADD (FADD without DED), pLXSN-cFLIP_L_ WT, pCR3-MC 159 WT and mutated MC159 (FLIP-DM; an analogous of cFLIP_L_ unable to activate NF-κB signaling). The amino acids that were mutated in each construct are highlighted with red. HEK 293T cells were transfected with mentioned plasmids (wt FADD, FADD-SLT2, FADD-DD, wt pEGFP-p65, pECFP-IKBα, FLIP-DM and wt cFLIP_L_). After 48 h, cells were harvested to monitor the (**b**) NF-κB luciferase reporter activity and (**c**) The expression of p65 and cFLIP_L_ by Western blot analysis, control represents vector transfected cells. The uncropped full-length blots are presented in supplementary Fig. S7. Error bars represent mean ± SD; In (**b**) *P ≤ 0.05, control vs mutant transfected cells (student t-test, n ≥ 3, where n is the number of independent experiments). wt = wild type.

Next, we used mutants of FADD and cFLIP_L_ to investigate the importance of individual domains to validate NF-κB activity and cFLIP_L_ expression. As mentioned in Fig. 3a the mutations created within each domain were marked in red in comparison to the wild type (wt) FADD and wt cFLIP_L_. All the constructs were transfected in HEK 293T cells for 48 h. We found that transient expression of FADD-DD (FADD without DED) and FADD-SLT2 (inactive DED^32^) showed elevated NF-κB activity, which was comparable to the p65 and wt cFLIP_L_ expressed cells. This suggests that DD of the FADD interacts with a DD of death receptors to block downstream apoptotic cascade to support pro-survival NF-κB signaling (Fig. 3b). Notably, mutant FLIP (DM-FLIP^35^) did not show major changes in NF-κB activity, p65 and cFLIP_L_ expression as compared to wt cFLIP_L_ and p65 expressed cells (Fig. 3b,c). Moreover, the apoptotic death analysis with the mutants showed no remarkable alteration in cell viability as compared to the cells expressing death inducer IκBα (Fig. S6a,b). Altogether, these results suggest that integrity of individual domains of FADD and cFLIP_L_ are essentially required to regulate NF-κB activation and apoptotic signaling response.

### FADD induces apoptosis independent of TNF-α stimulation

**Figure 4:**
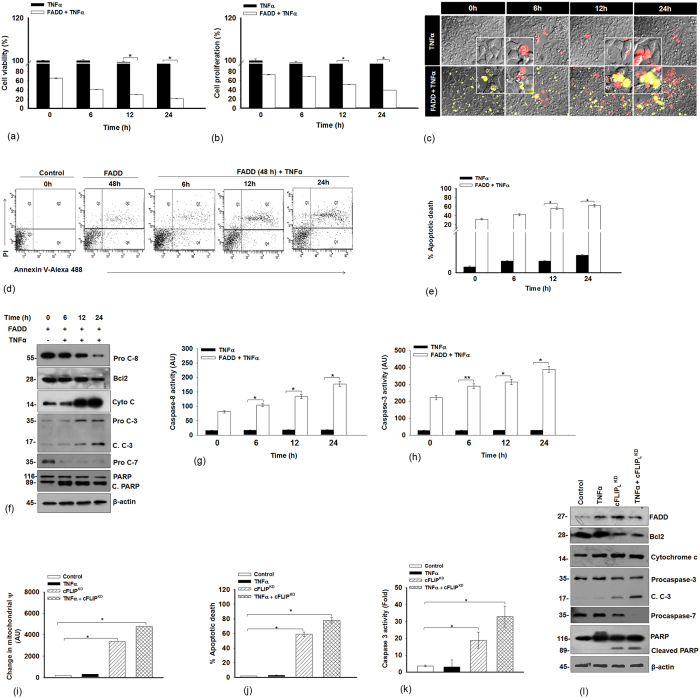
FADD augments downstream apoptosis signaling in TNF-α stimulated cells. TNF-α (10 ng/ml) was subjected to HEK 293T cells and 48 h of pcDNA-FADD transfected HEK 293T cells. Control represents HEK 293T cells without TNF-α treatment (black bar) and 48 h pcDNA-FADD transfcted HEK 293T cells (white bar) (shown as 0 h time point). (**a**) Percent cell viability, (**b**) Percent cell proliferation, (**c**) Images of Propidium iodide (PI) staining (pEYFP-FADD construct was used in this experiment) (scale bar-2 μm), (**d**) Apoptotic cell death monitored by Flow cytometric analysis using (BD FACSAria 3, BD Biosciences, San Jose, CA, USA BD), the result represents in contour plots with quadrant gates showing early apoptotic shown in quadrant 4 (Q4) and late apoptotic in quadrant 2 (Q2), (**e**) Percent apoptotic death (Annexin-V-FITC^+^/PI^+^) by Tali^TM^ image based cytometer, (**f)** Expression of of cell death regulatory proteins examined by Western blotting, **(g)** caspase-8 activity, (**h**) caspase-3 activity. The uncropped full-length blot of Procaspase-8, processed caspase-3 and cytochrome c are presented in supplementary Fig. S10. Next, HEK 293T cells were treated as mentioned in Figure legend 2i, TNF-α untreated and non targeting siRNA transfected cells were taken as control. Here results illustrate (**i)** Quantitative analysis of loss of mitochondrial membrane potential (Ψ), (**j**) Percent apoptotic death (Annexin-V-FITC^+^/PI^+^) by Tali^TM^ image based cytometer, (**k**) Caspase-3 activity, (**l**) Representative images of Western blot for cell death regulatory proteins. The uncropped full-length blot of FADD is presented in supplementary Fig. S10. Note that, the y axis break in (**a**,**b**) indicates the scale has been compressed between 97–99% and in (**e**) scale has been compressed between 7–10%. Error bars represent mean ± SD; In (**a**,**b**,**e**,**g**,**h**), *P ≤ 0.05 & **P ≤ 0.001, TNF-α treatment in non-transfected vs FADD transfected cells (One way ANOVA followed by Student Newman-Keuls test, n = 4), In (**i**–**k**), *P ≤ 0.05, control vs TNF-α or cFLIP_L_^KD^ or TNF-α + cFLIP_L_^KD^, (student t-test, n = 4), where n is the number of independent experiments.

Here, we demonstrate that FADD diminishes the expression of cFLIP_L_ and NF-κB activation, independent of TNF-stimulation. We showed the downstream signaling of cell death in FADD expressed cells subjected to TNF-α. First, we examined the cell viability with different concentration of TNF-α (5, 10 and 15 ng/ml) in HEK 293T cells. 2–3% of cell death was found post 24 h of incubation in 10 and 15 ng/ml TNF-α treated cells respectively (Fig. S8a–e). Furthermore, cell viability, cell proliferation and colony formation assay were carried out in non-transfected and 48 h of FADD expressed HEK 293T, HCT 116 and MCF-7 cells followed by TNF-α (10 ng/ml) exposure for 6–24 h. We did not find any major changes in the cell viability, proliferation and colony formation upon TNF-α treatment to non-transfected cells. However, FADD expressed cells treated with TNF-α abolishes the cell viability, cell proliferation and colony forming abilities (Fig. 4a,b; Figs S8f,g and S9a,b). Further, mode of cell death was confirmed by porpidium iodide (PI) staining, flow cytometry and Tali image based cytometer. We observed that TNF-α treatment to FADD expressed HEK 293T cells showing a gradual increase of PI positive cells and percent of apoptotic cell death as compared to the non-tranfected and untreated cells (Fig. 4c–e; Fig. S9c,d). Next, we monitored the mitochondrial integrity by transfecting FADD in the GFP-cytochrome c expressing HEK 293T cells and further stimulated with TNF-α for an additional 6–24 h. We noticed a progressive loss of mitochondrial integrity with simultaneous cytosolic accumulation of cytochrome c and depletion of Bcl-2 protein expression (Fig. 4f; Fig. S9e,f). More importantly, FADD expressed cells exposed to TNF-α showed activation of initiator procaspase-8, executioner caspase-3, procaspase-7 with cleavage of PARP as well as caspase-8 and caspase-3 activity (Fig. 4f–h). Subsequently, we analyzed the possibilities of necrotic cell death. The Necrostatin-1 pre-treated cells were transfected with pcDNA-FADD and further stimulated with TNF-α. We did not find release of LDH (a marker of necrosis) from the FADD expressed cells stimulated with TNF-α and the cleavage pattern of PARP, confirming that mode of cell death with the aid of FADD was apoptosis rather than necrotic (Fig. S11a,b). Further, we investigated the apoptotic consequences in cFLIP_L_ knockdown cells primed with TNF-α in HEK 293T cell. We noticed that knowckdown of cFLIP_L_ also attributes intrinsic signaling of cell death astonishingly with the concurrent release of cytochrome c and downregulation of Bcl-2 accompanied by activation of caspase-3, caspase-7 and cleavage of PARP (Fig. 4i–l; Fig. S9g–i). Altogether, these results confirm that FADD transduces apoptotic cell death signaling independent of TNF-α stimulation.

### FADD interacts with RIP1 by inhibiting the expression of cIAP2

**Figure 5:**
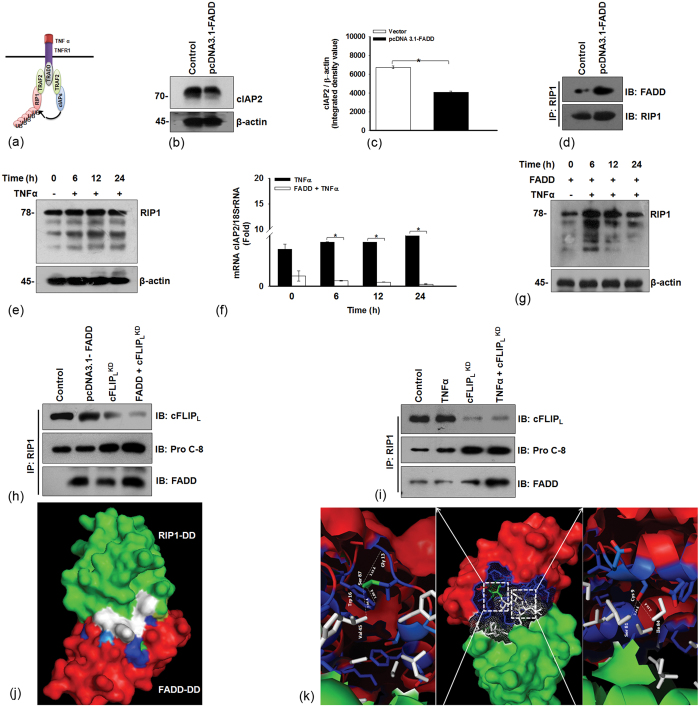
FADD abrogates cIAP2 expression and interacts with RIP1 and procaspases-8. (**a**) Illustration represents cIAPs mediated regulation of RIP1 at complex I. (**b**) HEK 293T cells were transfected with pcDNA-FADD for 48 h and then cells were harvested and subjected to western blot analysis to examine the expression of cIAP2 and (**c**) Densitometry (IDV- Integrated density value) of cIAP2 immunoblot. (**d**) Co-immunoprecipitation analysis of FADD and RIP1 on 48 h of FADD transfected HEK 293T cells. Control represents vector transfected cells. (**e**) HEK 293T cells were treated as mentioned conditions in the figure legend 2b and expression of RIP1 was examined by Western blotting. (**f**) HEK 293T cells were treated as mentioned in the figure legend 2c and mRNA expression of cIAP2, (**g**) Western blot of RIP1. (**h**) HEK 293T cells were treated as mentioned in the figure legend 2 g, control represents pcDNA 3.1 vector and non targeting siRNA transfected cells that were subjected to co-immunoprecipitation analysis of RIP1 with the mentioned antibodies. (**i**) HEK 293T cells were treated as mentioned in the figure legend 2i, control represents untreated and non targeting siRNA transfected cells that were subjected to co-immunoprecipitation analysis of RIP1 with the mentioned antibodies. (**j**) The possible interaction of FADD-RIP1 shown by the molecular docking model, (**k**) FADD-RIP1 interaction represents that Trp16 and Val45 were located on α2 and α4 helix of FADD-DD respectively and interacts with Ser 87 located on the α5 helices of RIP 1 DD; Cys9 located on α1 helix of FADD-DD interacts with Ser81 and Ile84 located on α5 helices of RIP 1-DD, H-bond in angstrom (Å) denoted with lines. The uncropped full-length blots of Fig. (**g**,**i**) are presented in supplementary Fig. S13. Error bars represent mean ± SD; In (**c**), *P ≤ 0.05 Vector transfected vs FADD transfected, (student t-test, n ≥ 3), In (**f**), *P ≤ 0.05 TNF-α treated untransfected vs TNF-α treated FADD transfected, (student t-test, n = 4), where n is the number of independent experiment.

Although, it is known that TNF-α triggers signaling of apoptotic cell death *via* formation of complex II^36^. Indeed, the formation of a DD complex between FADD, RIP1 and caspase-8 remains under the surveillance of anti-apoptotic proteins cIAPs and cFLIP^37^,^38^,^39^. As shown above FADD regulates NF-κB activation and cFLIP expression. Therefore, next, we were desired to investigate the involvement of FADD in the regulation of cIAPs (Fig. 5a). First, we analyzed the expression of cIAP2 in FADD expressed HEK 293T cells. We found that transient expression of FADD remarkably reduces the expression of cIAP2 in HEK 293T cells (Fig. 5b,c). Next, the co-immunoprecipitation analysis of FADD and RIP1 showed that mitigating the expression of cIAP2 propels the interaction of RIP1 with FADD (Fig. 5d). Previously, it has been shown that, the formation of RIP1 mediated complex II is tightly regulated by the process of ubiquitination^40^. In this context, we examined the ubiquitination of RIP1 during TNF-α stimulation. As anticipated, TNF-α stimulation instigates degradation of RIP1 as observed by smearing and laddering pattern with the progression of time from 6 h onwards (Fig. 5e). In contrast, induced expression of FADD downregulates the mRNA levels of cIAP2 and reduces the degradation of RIP1, which indicates that FADD protects RIP1 even in the presence of TNF-α with subsequent activation of procaspases 8 (Figs 4f,g and 5f,g). In addition, transient expression of FADD in HEK 293T cells alters the mRNA levels of cFLIP_L_ and upregulates the expression of FADD, RIP1 and procaspase-8 independent of TNF-α stimulation (Fig. S12a–d). Furthermore, we knocked down the expression cFLIP_L_ by siRNA in FADD expressed cells to find out whether the relieving cFLIP_L_ expression allows interaction of RIP1 with FADD and procaspase-8. The co-immunoprecipitation analysis suggests that down regulation of cFLIP_L_ by inducing expression of the FADD *(lane 2)*, and knockdown of cFLIP_L_ by siRNA *(lane 3)* as well as co-expression of FADD and cFLIP-siRNA *(lane 4)* showing sustained interaction of RIP1 with procaspase-8 *(row 2)* and FADD *(row 3)* (Fig. 5h). Next, we stimulated HEK 293T cells with TNF-α to stabilize complex I and further knockdown the expression of cFLIP_L_. We noticed that transient silencing of cFLIP_L_ favors RIP1-FADD interaction *(lane 3).* Interestingly, priming of TNF-α with simultaneous knockdown of cFLIP_L_ *(lane 4)* profoundly drives FADD-RIP1-caspase-8 associated complex II formation (Fig. 5i). Furthermore, the in-depth analysis of FADD driven stability to RIP1 and their binding interaction were analyzed by *in silco* approach. The molecular models of DD of FADD (FADD-DD) and RIP1-DD were constructed and molecular docking was performed by GRAMMX software^41^. *In silico* analysis of protein-protein interaction (PPI) suggests that involvement of a large number of hydrogen bonding along with other intermolecular interactions (such as electrostatic and vanderwaals) and larger contact interface provides a strong confirmation stability and specificity towards molecular recognition of FADD-DD by RIP1-DD and *vice –versa* (Fig. 5j,k). All together, these data corroborate that FADD stabilizes RIP1 and complex II by neutralizing the expression of cIAP2 and cFLIP_L_.

### FADD promotes JNK1 mediated ubiqutination of cFLIP_L_ to augment apoptosis

**Figure 6:**
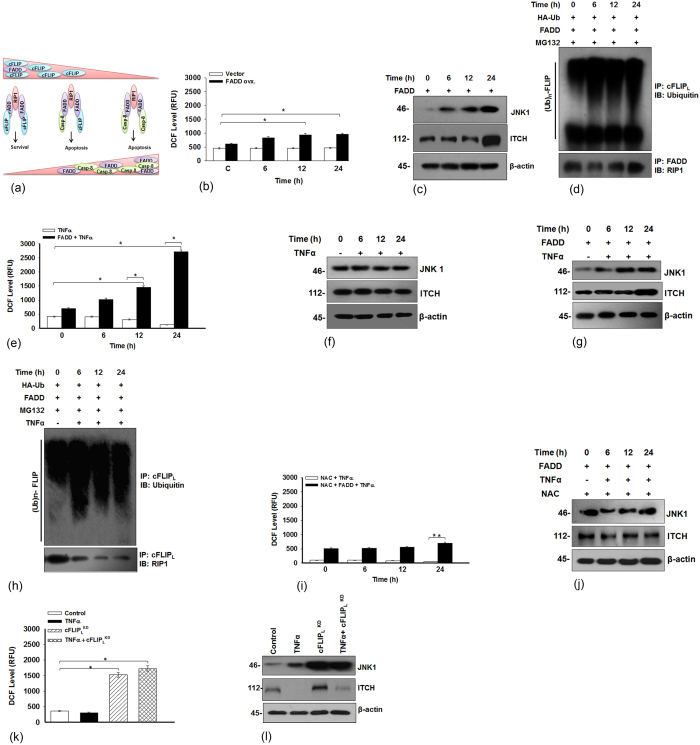
FADD triggers JNK1 mediate ubiquitination of cFLIP_L_. (**a**) Illustration represents FADD stabilizes RIP1 and procaspase-8 associated pro-apoptotic complex II. (**b**) HEK 293T cells were transfected with pcDNA3.1-FADD for mentioned time points, control represents 48 h of FADD (shown as 0 h time point) expressed cells, to examine the cellular ROS, (**c**) Expression of JNK1 and ITCH and (**d**) The ubiquitination of cFLIP_L_ in MG132 (10 μM for 3 h) pre-treated cells subjected to co-immunoprecipitation analysis. From the same cell lysate the co-imunoprecipitation of FADD with RIP1 was carried out. (**e**) HEK 293T cells were treated as mentioned in the figure legend 2c to examine the level of cellular ROS and (**f**) Expression of JNK1 and ITCH (**g**) HEK 293T cells were treated as mentioned in figure legend 2c and expression of JNK1 and ITCH (**h**) The 48 h pcDNA3.1-FADD transfected HEK 293T cells pre-treated with MG132 (10 μM for 3 h) followed by treatment of TNF-α (10 ng/ml) for mentioned time points and subjected to Co-IP assay for monitoring the ubiquitination of cFLIP_L_ and expression of RIP1. (**i**) The 48 h pcDNA3.1-FADD transfected HEK 293T cells pre-treated with N-acetyl cysteine (NAC) (25 μM for 3 h) followed by treatment of TNF-α (10 ng/ml) for mentioned time points and level of cellular ROS and (**j**) Expression of JNK1 and ITCH (**k**) HEK 293T cells were treated as described above in the figure legend 2i to determine the level of cellular ROS and (**l**) Expression of JNK1 and ITCH. The uncropped full-length blots of Fig. (**h**,**j**) are presented in supplementary Fig. S15. Error bars represent mean ± SD, in (**b**) *P ≤ 0.05, Vector transfected vs FADD transfected, (student t-test, n ≥ 3), in (**e**) *P ≤ 0.05, TNF-α treated untransfected vs TNF-α treated FADD transfected, (student t-test, n ≥ 3), in (**i**) **P ≤ 0.001, NAC and TNF-α treated, untransfected vs NAC and TNF-α treated, FADD transfected, (student t-test, n ≥ 3), in (**k**) *P ≤ 0.05, control vs TNF-α or cFLIP_L_^KD^ or TNF-α + cFLIP_L_^KD^, (student t-test, n ≥ 3), where n is number of independent experiments.

We demonstrate here that FADD stabilizes RIP1 and procaspase-8 associated complex II as shown in illustration (Fig. 6a). Although previous studies have shown that the suppression of NF-κB and cFLIP accumulates cellular ROS and activates JNK1 dependent cell death^28^,^42^,^43^. Moreover, activation of JNK1 has been shown to regulate the expression of cFLIP_L_ by activating ubiquitin ligase ITCH^26^. The results shown above clearly demonstrated that FADD plays dominating role in abolishing the expression of cFLIP_L_ to execute apoptotic cell death. Next, we were interested to investigate the underlying mechanism of FADD mediated turnover of cFLIP_L_ in HEK 293T cells. We observed that induced expression of FADD accumulates intracellular ROS with the simultaneous activation of JNK1 and E3 ubiquitin ligase ITCH (Fig. 6b,c). Moreover, mechanistically we found a novel outcome that, FADD induces time dependent ubiqutination of cFLIP_L_ and strongly interacts with RIP1 to turn over the expression of cFLIP_L_ (Fig. 6d). Thus, these findings would be a possible explanation for our earlier results exemplifying successive loss of cFLIP_L_ from the DISC. This outcome prompted us to investigate the expression of JNK1 and ITCH in TNF-α treated HEK 293T and FADD expressed HEK 293T cells. We found that exposure of TNF-α alone to HEK 293T suppresses intracellular ROS and had a negligible effect on the expression of JNK1 and ubiquitin ligase ITCH (Fig. 6e,f). Surprisingly, exposure of TNF-α to FADD expressed cells showing accumulation of ROS with subsequent activation of JNK1 and ITCH mediated ubiquitination of cFLIP_L_ (Fig. 6e,g,h). However, in the presence of ROS scavenger N-acetyl cysteine (NAC) cells restore JNK1 and ITCH expression as similar to the control cells (Fig. 6i,j). indicating that FADD promotes ROS and JNK1 mediated ubiquitination of cFLIP_L_ to augment apoptotic cell death. In addition, the knockdown of cFLIP_L_ in TNF-α pre-stimulated cells showed a drastic elevation in ROS generation and ITCH activation (Fig. 6k,l), but pre-treatment with NAC, abolishes JNK1 and ITCH activation (Fig. S14a,b). Altogether, these results indicate that FADD plays an important role in turnover the expression of cFLIP_L_ by inducing ROS dependent activation of JNK1 and ubiquitin ligase ITCH.

## Discussion

In this study, we describe the molecular mechanism of programmed cell death signaling coordinated by FADD in context to TNF-α stimulation. The cell death receptor signaling is predominantly regulated by pro-apoptotic functional protein FADD and caspases-8 that initiates downstream signaling of apoptosis^7^,^44^,^45^. Although, previous reports suggest that FADD also participates in non-apoptotic function including embryonic development and T-cells proliferation apart from their apoptotic function^6^,^46^,^47^. Interestingly, dual role of FADD in cell death and survival remains controversial. Mechanistically, the adaptor protein FADD provides a common platform for binding of procaspase-8 and cFLIP_L_ to regulate death receptor mediated apoptosis^48^. As death receptor mediated apoptosis is inhibited by cFLIP, which helps to maintain tissue homeostasis by protecting the outcomes of cell death with the aid of NF-κB activation^30^. Accumulating evidence have advocated that, low expression of FADD and elevated expression of cFLIP_L_ restricts apoptosis and promotes cell proliferation^49^,^50^. In this respect, we examined the expression of FADD and cFLIP_L_ in different origin of cell lines. We found that expression of FADD was minimal in MCF-7, HeLa, HEK 293T and HaCaT cells, but expression of cFLIP_L_ was high. In contrast, HCT 116, RAW 264.7 and A549 cells showed inadequate expression of FADD and aberrant expression of cFLIP_L_. Our previous findings revealed that induced expression of FADD in HEK 293T cells alleviates the expression of cFLIP_L_ and activate caspase-8 mediated apoptosis, independent of Fas ligand stimulation^27^. Consistent with these finding, we examined the interaction of cFLIP_L_ and pro-caspase-8 during availability of FADD. We observed that the added expression of FADD in HEK 293T cells dismantles the integrity of cFLIP_L_ and selectively interacts with procaspase-8 rather than cFLIP_L_ at the DISC.

Moreover, the major role ascribed to TNF-α for cell survival has been orchestrated by NF-κB activation and up regulation of anti-apoptotic proteins such as cFLIP, cIAPs, XIAP etc. that appears to prevent apoptosis signaling^7^. Paradoxically, TNF-α contributes cell death or survival *via* participating various signaling depending upon cellular context and stimulation^12^. Although, cFLIP_L_ advances TNF-α mediated MAPK/ERK1 and NF-κB activation for cell survival^34^. While, some reports suggest that cell death by apoptosis or survival by activation of NF-κB may depend upon the level of FADD, cFLIP and caspases-8^7^,^51^. In contrast, previous report suggests that FADD, casper and caspases-8 can activate NF-κB, whereas procaspase-8 mediated activation of NF-κB is independent of the proteolytic activation of caspases-8 that may precede for only apoptotic cell death^52^. Nevertheless, it has been also suggested that low dose of FADD contributes to activation of NF-κB, which is mainly relying on concentration and time dependent manner, but mutating the caspase-8 neutralizes this activation^53^. Although, activation of NF-κB signaling up regulates the expression of c-FLIP and *vice versa* to abrogate the activation of caspases-8^11^,^13^. Therefore, we were interested to unravel the cellular signaling of FADD in context of cell death or survival stimulation by TNF-α and NF-κB activation. Here, we found that TNF-α canonically induces NF-κB activation and associated gene product cFLIP_L_ in HEK 293T cells, but in the presence of FADD the expression of p65 and cFLIP_L_ was abrogated. In further agreement with NF-κB activation induced by TNF-α, we found that ectopic expression of FADD impedes NF-κB activation and obstructs cytosolic to nuclear translocation of p65. In support, we found that FADD ubiquitinates IKKβ and stabilize IκBα to canonically inhibits the nuclear translocation of p65. Thus, these results indicate that the stimulation of TNF-α in the presence of FADD failed to maintain the anti-apoptotic integrity of NF-κB and cFLIP_L_ for cell survival. Thus, FADD has ability to suppress NF-κB activation and induction of apoptosis. It might be possible due to the sufficient availability and concentration resulted cell death. Next, we examined the importance of constituting structural domains of FADD and cFLIP_L_ that are importantly associated with NF-κB activation and cFLIP_L_ expression. The transient expression of DD of FADD (FADD-DD) and mutant of the FADD (FADD-SLT2; that carries an amino acid alteration in the DED region of FADD^32^) shows a significant increase in the activity of NF-κB and expression of p65 and cFLIP_L_. These outcomes suggest that, the DD of FADD binds to DD of death receptors (DRs) and block the downstream apoptosis signaling to favor NF-κB activation and cell proliferation. Moreover, the ectopic expression of mutant cFLIP_L_ (DM-cFLIP_L_; have impaired TRAF2 binding domain required for NF-κB activation^35^) has negligible effect on the NF-κB activity, p65 and cFLIP_L_ expression, as compared to the conventional response generated from wild type cFLIP_L_. Thus above outcomes suggest that availability of FADD supports apoptotic association rather than NF-κB activation.

Next, we demonstrate here the potential of FADD on apoptotic commencement in TNF-α stimulated cells. We revealed that induced expression of FADD activates both extrinsic and intrinsic caspases responsible for apoptotic cell death, even in the presence of TNF-α. Surprisingly, TNF-α could not protect the cell survival during the availability of FADD in HCT 116 and MCF-7 cells. Similarly, pre-treatment of TNF-α followed by knockdown of cFLIP_L_ was unable to protect NF-κB activation and cell survival. In addition, we confirmed necrotic cell death and did not find the release of LDH and inhibition of PARP activation in FADD expressed cells pre-treated with necrostatin-1 followed by TNF- α exposure. Thus, our results clearly suggest that FADD attenuates NF-κB and cFLIP_L_ activation to restrict cell survival even in the presence of stimulant TNF-α. More interestingly FADD mediated proteolytic cleavage of pro-caspase-8 confers downstream signaling of apoptosis. As described earlier, TNF-α triggers the assembly of complex I constituted with RIP1/TRADD/TRAF2/cIAPs to activate NF-κB and downstream anti-apoptotic signaling network for cell survival^7^. However, depletion of cIAP1/2 relieves RIP1 from complex I to form pro-apoptotic complex II along with FADD and procaspase-8^39^,^54^. It has been shown that, TNF-α also triggers signaling of cell death *via* formation of complex II, comprising with FADD, procaspases-8 and RIP1 to execute apoptosis^55^. Moreover, TNF-α mediated cell death and survival decision is critically regulated by cIAPs dependent ubiquitination of RIP1 as an initial checkpoint and later on sheltered by cFLIP_L_^39^. Consistent with the previous findings, we were interested to explore the cellular dynamics of FADD in the regulation of cIAP2 expression and ubiquitination of RIP1 in response to TNF-α. The mRNA and protein expression analysis along with co-immunoprecipitation assay revealed that transient expression of FADD negatively regulates the expression of cIAP2 and favors the interaction of RIP1 with FADD. Moreover, selective knockdown of cFLIP_L_ in FADD expressed cells greets strong interaction between RIP1 and FADD. Similarly the mRNA analysis suggests that ectopic expression of FADD upregulated expression of caspase-8, however the expression of anti-apoptotic genes such as *cFLIP*_*L*_ and *cIAP2* were found depleted, independent of TNF-α stimulation. Thus, these results demonstrate that FADD has fine tuning over regulation of cIAPs, RIP1 and cFLIP_L_ even in the presence of pro-survival factor TNF-α. In addition, the *in silico* analysis suggests that the multiple bonding interaction and occupied larger surface area helps to provide a holistic environment for the catalytic interaction of FADD and RIP1 to form complex II.

In contrast, the cell death and survival signaling associated with NF-κB and cFLIP has strong co-relation with ROS production^56^,^57^. Previously, it has been shown that, ablation of NF-κB activation induces rapid generation of ROS and JNK activation in response to TNF-α^18^,^20^,^58^. Nevertheless, JNK activation is a highly regulated process. as short JNK1 activation favors tissue regeneration, whereas prolonged JNK activation promotes cell death^59^,^60^. Although, it has been earlier shown that activation of JNK orchestrates the E3 ubiquitin ligase ITCH mediated turnover of cFLIP_L_ expression^26^,^61^. Therefore, we were prompted to delineate the precise molecular mechanism of FADD mediated turnover of cFLIP_L_. Our results demonstrate that, induced expression of FADD accumulates ROS and activates JNK1 to trigger the ITCH mediated ubiquitination and degradation of cFLIP_L_. In contrast, stimulation of TNF-α alone does not show any major changes in the expression of JNK1 and ITCH. However, in the presence of FADD, stimulation of TNF-α significantly enhances ROS level and ubiquitination of cFLIP_L_ in HEK 293T cells. Moreover, knockdown of the cFLIP_L_ has been reported to augment ROS generation and JNK activation in tumor cells^28^,^62^. We demonstrate here that knockdown of cFLIP_L_ in TNF-α primed cells progressively enhance the ROS accumulation and JNK1 activation, but this effect was counterbalanced in NAC pre-treated cells. Thus, these results clearly indicate that ROS mediated activation of JNK1 and ITCH is also dependent on the expression of FADD and cFLIP_L_.

**Figure 7:**
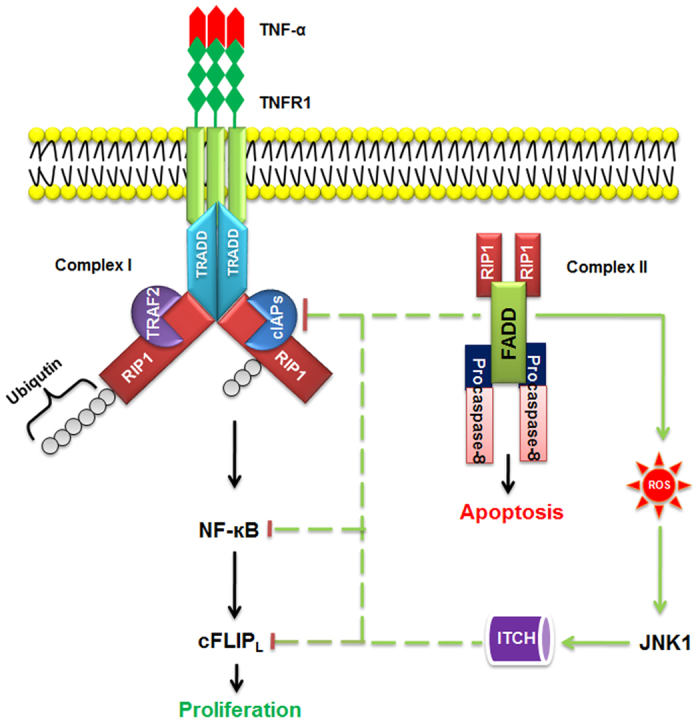
Illustration of FADD mediated regulation of TNFR1 complex for initiation of cell death. Ligation of TNF-α to TNFR1 induces its oligomerization and recruitment of adaptor protein TRADD, TRAF2, cIAPs and RIP1 to form complex I. The ubiquitin ligases TRAF2 and cIAPs allow ubiquitination of RIP1 to facilitate NF-κB activation and downstream transcriptional activation of cFLIP_L_ to cell survival. FADD simultaneously regulates cIAP2 expression and interacts with RIP1 along with procaspases-8 to form complex II for the initiation of cell death. FADD negatively regulate NF-κB activation and cFLIP_L_ expression. Additionally, FADD triggers ROS generation to activate JNK1 mediated ubiquitination of cFLIP_L_ for the commencement of apoptotic cell death.

In conclusion, this study delineates a novel molecular mechanism of FADD that regulates NF-κB activation and promotes JNK1 dependent ubiquitination of cFLIP_L_ to induce apoptosis, independent of TNF-α response as shown in illustration (Fig. 7). The balanced expression of FADD and cFLIP_L_ is important to determine the fate of cell death or survival. Thus, we can suggest that FADD is an important functional component in apoptosis signaling and it could be a plausible novel candidate for cancer therapeutic.

## Materials and Methods

### Chemicals

Molecular biology grade reagents were purchased commercially. Poly-L lysine, protease inhibitor cocktail, H_2_DCFDA, 2-(4-Amidinophenyl)-6-indolecarbamidine dihydrochloride, 4′,6-Diamidino-2-phenylindole dihydrochloride (DAPI), Thiazolyl Blue Tetrazolium Blue (MTT), Fluoromount, BCA protein estimation kit and anti-ITCH antibody (SAB4200036) were purchased from Sigma-Aldrich (St. Louis, MO, USA). Annexin V-FITC Apoptosis detection kit, Caspase-8/FLICE fluorometric assay kit, Necrostatin-1, Sepharose-A beads and JC-1 dye were purchased from BioVision, (Mountain View, California). Dulbecco’s Modified Eagle’s Medium (DMEM), Dulbecco’s Phosphate buffer saline (DPBS), Fetal bovine serum (FBS), Prestoblue viability assay kit, Lipofactmine, RNAi-MAX transfection reagent, MitoTracker Red CMXRos dye, Caspase-3 assay kit, anti-JNK1(44-690G), anti-Bcl2 (138800), anti-mouse HRP linked secondary antibody (S921) and Alexa fluor 635 were purchased from Invitrogen (Life Technologies, USA). Dual glow luciferase assay kit was purchased from Promega (Madison, WI, USA). Rabbit polyclonal antibodies against p65 (8242P), Procaspase-9 (9502S), Procaspase-3 (9664P), Procaspase-7 (9492P), Cytochrome c (4272S), PARP (9542P), RIP1 (3493), β-actin (4967S) and anti-rabbit HRP linked secondary antibody (7074P2) were purchased from Cell Signaling Technology (Danvers, MA). cFLIP_L_ (NBP1-45479) and FADD (NB100-92032) were purchased from Novus Biological (USA). LDH Cytotoxicity Detection Kit (MK401) was commercially purchased from TaKaRa-Clontech (USA). TNF-α was purchased from ProSpec (Israel). Anti-Ubiquitin (P4D1) was purchased from Enzo life sciences (USA). Anti-cIAP2 (552782) and anti-caspase-8 (551242) were purchased from BD Pharmigen (USA). Anti-IKKβ (ab32135) was purchased from Abcam (USA). EZ Blue^Tm^ cell assay kit was purchased from HiMedia (Mumbai, India). All other chemicals used were of analytical grade and purchased from Merck (Darmstadt, Germany).

### Cell lines and culture

HEK 293T, NIH 3T3, RAW 264.7, HCT 116, HaCaT cell lines were obtained from ATCC (Manassas, VA, USA). MCF-7, HT-29, HeLa and A549 were obtained from NCCS, Pune India. HEK 293T, MCF-7, HeLa, HaCaT and NIH 3T3 cells were grown in DMEM culture media containing L-glutamine (2 mmol/l). HT-29, RAW 264.7, HCT 116, and A549 cells were grown in RPMI-1640 culture media containing L-glutamine (2 mmol/l). All the media were supplemented with 10% fetal bovine serum and an antibiotic cocktail containing penicillin (5 mg/ml), streptomycin (5 mg/ml) and neomycin (10 mg/ml) (GIBCO, Invitrogen, UK). The cells were kept in a humidified atmosphere of 95% O_2_ and 5% CO_2_ in a CO_2_ incubator at 37 °C. Exponentially growing cultured cells were used for further experiments.

### Plasmid constructs and transfection

Plasmid expression vectors encoding pEYFP-FADD (provided by Dr. Andrew Thorburn, University of Colorado), pEGFP-p65, HA-Ub and pECFP-IκBα (provided by Dr. J A Schmid, Medical University, Viena), pcDNA3.1, pcDNA-FADD, FADD-SLT2 and FADD-SLT4 (provided by Dr. Penelope Duerksen-Hughes, Loma Linda University, USA), pNFκB-Luc : pGL3b-kB4/pRL-TK : Renilla Luciferase (provided by Dr. Susan Nozell, University of Alabama at Birmingham), pLXSN-cFLIP_L_ (provided by Prof. Sabine Adam, Universitätsklinium Schleswig-Holstein, Germany), pCR3-MC159 (cFLIP_L_) and DM-cFLIP_L_ (provided by Prof. Margot Thome, USA), pEGFP-cytochrome-c (provided by Dr. Douglas Green, St. Jude Children’s Research Hospital, USA) and pmCherry-BID (provided by Dr. Eyal Gottlieb, Cancer Research UK Beatson Institute) were transfected into HEK 293T, MCF-7 and HCT 116 cells using lipofectamine LTX plus transfectiont reagent (Life Technologies, USA), according to manufacturer’s instructions.

### Knockdown of cFLIP_L_ by siRNA

**s**iRNA oligonucleotide targeted against cFLIP_L_ was custom synthesized from Invitrogen, (Life Technologies, USA). In brief, HEK 293T cells were seeded at a density of 5 × 10^5^ cells in a 6 well plate and incubated for 16 h followed by transfectetion of siRNA-cFLIP_L_ (75 nM) along with 7.5 μl of RNAiMAX™ (Invitrogen, Life Technologies, USA) in a total volume of 200 μl per well in serum free OptiMEM culture media (Life Technologies, USA). Cells were incubated with siRNA-cFLIP_L_-RNAiMAX™ for further 48 h. The non targeting siRNA was taken as a negative control.

### ROS measurement

To determine the level of ROS, HEK 293T cells were plated at a density of 2 × 10^4^ cells per well in a black bottom 96 well plates and incubated in a CO_2_ incubator for 24 h. Cells were subjected to treatments as described in the figure legends. The total ROS was determined using a fluorescent dye 2′, 7-dichlorodihydrofluoroscein-diacetate (H_2_DCF-DA) as described earlier^63^. The H_2_DCF-DA intracellularly turns to highly fluorescent DCF molecule upon oxidation. The result represents intracellular DCF level.

### Cell Viability, proliferation and Apoptotic Cell Death Analysis

The percent cell viability and percent cell proliferation were evaluated by Prestoblue cell viability kit (Life Technology, USA), MTT assay and EZ^TM^ blue cell assay kit (HiMedia, India) according to the manufacturer’s instructions. In brief, HEK 293T cells were seeded at a density of 2 × 10^4^ cells in a 96 well plate and incubated for 24 h. Cells were subjected to treatments as described in the figure legends. MTT assay was carried out as described earlier^64^. Next, the percent apoptotic cell death was examined by Tali^TM^ image based cytometer (Life Technologies, USA) as described previously^63^.

### Flow cytometry analysis

The apoptotic cell death was also confirmed by flow cytometry (FACSAria 3, BD Biosciences, San Jose, CA, USA). In brief, HEK 293T cells were seeded at a density of 2 × 10^6^ cells in a 60 mm dish and incubated for 24 h. Cells were subjected to treatments as mentioned in the figure legends. Thereafter, cells were collected, washed and re-suspended in 1X Annexin binding buffer followed by the addition of Annexin-V-FITC and Propidium Iodide solution (BD Biosciences, New Jersey, USA). Cells were incubated in the dark for 20 min at room temperature and thereafter subjected to flow cytometric analysis. Data were acquired by BD FACSDiva software (BD Biosciences, San Jose, CA, USA) using standard fluidics, optical and electronic configuration. The light source used was blue laser 488 nm with filters, FITC (530/30) and PI (585/42). The FITC and PI channels were compensated with appropriate controls. The Gating on the cell population was set up by FSC/SSC scatter plot. 10,000 events were recorded and analyzed for Annexin-V/Propidium Iodide stains. The results represented in contour plots with quadrant gates showing early apoptosis in quadrant 4 (Q4) and late apoptosis in quadrant 2 (Q2).

### Propidium Iodide staining

Cellular integrity was validated by propidium iodide (PI) staining using an apoptosis detection kit (BioVision, USA). In brief, 1 × 10^5^ cells were grown on poly L-lysine coated coverslips kept in six-well plates for 24 h. Cells were subjected to treatments as described in the figure legends. Post incubations cells were stained with propidium iodide and images were captured under a fluorescent microscope (DP71, Olympus, Japan) in DIC and fluorescent mode, the result represents merged images of cells in DIC with PI stained nuclei. More than 200 cells from three different fields were analyzed.

### Colony formation assay

The colony formation assay was performed by crystal violet staining. In brief, HEK 293T cells were seeded at a density of 1 × 10^5^ cells per well and cultured overnight, followed by FADD transfection and TNF-α exposure for mentioning time points. The colony formation assay was performed as previously described^64^. The result represents percent crystal violet stained colony formation relative to control.

### p65 translocation assay

In brief, HEK 293T cells were seeded at a density of 1 × 10^5^ on a coverslip kept in 24 well plate and incubated for 24 h. Thereafter cells were transfected with pEGFP-p65 and expressed for an additional 24 h. Further cells were subjected to treatments as described in the figure legends. The cytosol to nuclear translocation of GFP tagged p65 was monitored under a fluorescent microscope (DP71, Olympus, Japan). More than 150 cells from three random fields were analyzed.

### NF-κB luciferase reporter assay

In brief, HEK 293T cells were seeded at a density of 1 × 10^5^ cells in a 24 well plate and incubated for 24 h. Thereafter, cells were co-transfected with pGL3b-kB4, a luciferase reporter plasmid and pRL-TK, a thymidine kinase promoter-Renilla luciferase reporter plasmid using lipofectamine LTX plus transfectiont reagent (Invitrogen, Life Technologies, USA) according to manufacturer’s instructions. Cells were subjected to treatments as described in the figure legends. The NF-κB luciferase reporter assay was performed with Luciferase Assay Kit (Promega, USA) as per manufacturer’s instructions. At the end of incubation, cells were lysed in 100 μl of lysis buffer and cell lysate was mixed with 1:5 ratio of luciferase assay reagent (LARII). The values of firefly and renilla were recorded in a Luminometer (Centro LB 960, Berthold, USA). The results are expressed as the ratio of firefly luciferase activity to that of renilla and normalized to protein concentration.

### Lactate Dehydrogenase (LDH) release assay

The release of LDH was analyzed by LDH cytotoxicity detection kit (Takara-Clontech, USA). In brief, HEK 293T cells were seeded at a density of 2 × 10^4^ cells in a 96 well plate and incubated for 24 h. Cells were subjected to treatments as described in the figure legends. Post incubation the culture medium was equally mixed with the reaction buffer and incubated for 30 min at room temperature in the dark. The absorbance was recorded at 490 nm with reference at 650 nm using a Multimode microplate reader (Molecular Devices, USA). The result represents percentage release of LDH.

### Isolation of RNA and Real Time-qPCR

Total cellular RNA was extracted from a mentioned cell line using Trizol reagent (Invitrogen, Life Technology, USA) and reverse transcribed into cDNA using the iScript cDNA Synthesis Kit (Bio-Rad Laboratories) as per the manufacturer’s instructions. The cDNA was then amplified and analyzed by RT-qPCR as previously described^27^. The primer sequences of respective genes are available upon request. Each assay was normalized by using the difference in critical thresholds (C_T_) between target genes and 18SrRNA. The expression of mRNA of respective genes was compared with control using the values of 2 ^−ΔΔCT^.

### Western blotting

In brief, cells were seeded at a density of 4 × 10^5^ cells in a 6 well plate and incubated for 24 h. Cells were subjected to treatments as described in the figure legends and processed for SDS-PAGE and western blotting as described previously^63^. In the present study, membrane was blocked with 5% non-fat milk in Tris buffered saline for 3 h at room temperature followed by overnight incubation with following primary antibodies including anti-FADD (1:1000), anti-cFLIP_L_ (1:500), anti- p65 (1:1000), anti-ITCH (1:500), anti-PARP (1:1000), anti-cytochrome c (1:500), anti-caspase-3 (1:1000), anti-procaspase-8 (1:200), anti-procaspasse-7 (1:500), anti-procaspase-9 (1:1000), anti-JNK1 (1:200), anti-Bcl2 (1:500), anti-Ubiquitin (1:1000), anti-RIP1 (1:1000), anti-cIAP2 (1:250), anti-IKKβ (1:500) and anti-β-actin (1:2000) at 4 °C. After washing with TBST, the membrane was probed with horseradish peroxidase conjugated secondary antibodies (1:10,000). Expression of immunoreactive proteins was detected by using Novex^®^ ECL HRP linked chemiluminiscent substrate kit (Invitrogen, USA) according to the instruction manual and developed in Kodak X-Omat blue film (NEN Life Sciences, Inc., Boston, MA) in the dark.

### Immunostaining

In brief, HEK 293T cells at a density of 1 × 10^5^ cells/well were seeded in 6-well plate containing cover slip and subjected to treatments as described in the figure legends. Further cells were fixed with 4% paraformaldehyde and permeabilized with 0.5% Triton X-100 in PBS at room temperature for 30 min. Cover-slips were then washed three times for 5 min each with PBS and then blocked for 1 h in 1% BSA at room temperature. Cells were then gently incubated with Primary antibody (anti-FADD & anti-cFLIP_L_) at a dilution of 1:50 for 16 h at 4 °C. Cover-slips were washed three times for 5 min each with TBS and incubated at room temperature for 1 h with Alexa fluor conjugated secondary antibody at a dilution of 1:100. The cells were counterstained with DAPI (1 μg/ml) for 5 min at room temperature. Cover-slip were then washed two times with TBS and mounted with Fluoromount mounting media. The images were visualized under the laser scanning confocal microscope (Leica TCS SP5 II, Germany).

### Co-immunoprecipitation analysis

To analyze the DISC and binding association of FADD or cFLIP_L_ to RIP1 during FADD expressed cells or cFLIP_L_ knockdown condition co-immunoprecipitation was carried out. In brief, 2 × 10^6^ HEK 293T cells were seeded in 60 mm dishes followed by treatments as described in the figure legends. The cells were lysed in 1 ml of lysis buffer (20 mM Tris-HCl, pH 7.4, 150 mM NaCl, 10% glycerol, 0.2% Nonidet P40, and 1X protease inhibitor mixture (Roche, Switzerland) for 30 min on ice. The total cell lysate was centrifuge at 15,000 g for 15 min at 4 °C. The supernatant was collected and protein concentration was determined by the BCA protein estimation kit (Sigma, USA). Next, 300 μg of protein was incubated with 20 μl of Protein A-Sepharose beads (BioVision, USA) for 1 h at 4 °C with gentle shaking. The mixture was spun down at 3000 rpm for 5 min at 4 °C and the collected supernatant was incubated with 1 μg of respective antibodies and 20 μl of Protein A-Sepharose beads for overnight at 4 °C with gentle shaking. The beads were washed three times with 1 ml of RIPA buffer (50 mM Tris-HCl (pH 7.4), 150 mM NaCl, 10% glycerol, 1% triton-X 100, 0.5% Na-deoxycholate, 0.1% SDS and 1X protease inhibitor mixture) and finally resuspended in 6X Laemmli sample buffer. The protein samples were fractionated on 12% SDS-PAGE followed by western blot as described above.

### Measurement of Caspase 8 and Caspase 3 activity

Caspase-8 activity was determined by Caspase-8/FLICE fluorometric assay kit (Biovision, U.S.A.) as previously described^27^. The result represents fold activity of caspases-8 with respect to control cells. Next, caspase-3 activity was determined by EnzChek Caspase-3 Assay Kit (Invitrogen, Life Technologies, USA) as previously described^63^. The results represented as fold increase in the activity of caspases-8 and caspase-3 with respect to control.

### Measurement of Mitochondrial membrane potential (ΔΨm)

Change in mitochondrial membrane potential (ΔΨm) was determined by using JC-1 fluorescent dye. In brief, HEK 293T cells were seeded at a density of 2 × 10^4^ cells per well in a 96 well plate and cultured for 24 h and then cells were subjected to treatments as described in the figure legends. The assay was performed as previously described^63^. The results represented as fold change in mitochondrial membrane potential with respect to control.

### Mitochondria staining

To examine the mitochondrial mass, the mitochondrial staining was performed using MitoTracker^®^ Red CMXRos dye (Invitrogen, Life Technologies, USA) according to manufacturer’s instructions. In brief, HEK 293T cells were seeded at a density of 1 × 10^5^ cells on a coverslip kept in 24-well plate and cultured for 24 h, further cells were subjected to treatments as described in the figure legends. The cells were stained with MitoTracker dye (100 nM) for 30 min in dark at 37 °C followed by counterstained with DAPI for 5 min in the dark and then covered with fluoromount mounting medium (Sigma-Aldrich, USA). The images were visualized under the laser scanning confocal microscope (Leica TCS SP5 II, Germany).

### Confocal microscopy

For confocal microscopy, 1 × 10^5^ HEK 293T cells were grown on poly-L lysine coated glass coverslips kept in a 24-well plate and allowed to incubate for 24 h. The adherent cells were subjected to treatments as described in the figure legends. Cells were washed with DPBS (pH7.4) and fixed with 4% paraformaldehyde at room temperature for 5 min and stained as mentioned earlier. Cells were washed with PBS and counterstained with DAPI for 5 min in the dark and covered with fluoromount mounting medium (Sigma-Aldrich, USA). The images were captured with a laser scanning confocal microscope (Leica SP5, Germany). All the images were further analyzed and processed with Leica SP5 II software (Leica TCS SP5 II, Germany).

### *In silico* protein-protein interaction

Unavailability of suitable crystallographically resolved structure for death domain of RIP1 (RIP1-DD; 583–669) and FADD (FADD-DD; 97–181) in the available structural databases, forced us to construct a molecular model of both by computational techniques. The 3D models of death domain (DD) of FADD (97–181; PDB ID: 2GF5) with 100% sequence identity and more than 99% confidence and DD of RIP1 (583–669; PDB ids: 2YQF, 2OF5, 1FAD, 1DDF, 4O6X, 1WXP) with a sequence identity ranging from 24–33% and more than 99% confidence level, were generated by protein modelling tools Phyre2^65^, ModWeb^66^, SWISS-MODEL^67^ and RaptorX^68^. The generated models of both death domain sequences were inspected with the help of CHIMERA^69^ and PyMol (The PyMOL Molecular Graphics System, Version 1.7.4 Schrödinger, LLC) structural tools. The best model was selected for each; based on the sequence identity, query coverage and confidence level of model building. The molecular docking between DD models of FADD and RIP1 was performed by program GRAMMX for unravelling protein-protein interaction (PPI)^41^.

### Statistical analysis

Statistical analysis was performed by one way analysis of variance (ANOVA) followed by Student Newman Keulas test for multiple comparison and Student t-test using SigmaStat statistical analysis software. Values were expressed as mean ± S.E.M. from three independent experiments. Differences were considered statistically significant at *P ≤ 0.05 and **P ≤ 0.001.

## Additional Information

**How to cite this article**: Ranjan, K. and Pathak, C. FADD regulates NF-κB activation and promotes ubiquitination of cFLIP_L_ to induce apoptosis. *Sci. Rep.* **6**, 22787; doi: 10.1038/srep22787 (2016).

## Supplementary Material

Supplementary Information
