# Supplementary material for: FADD regulates NF-κB activation and promotes ubiquitination of cFLIPL to induce apoptosis
Source: Sci Rep. 2016 Mar 14;6:22787. doi: 10.1038/srep22787 (PMC4789601; doi:10.1038/srep22787)
Supplement: Supplementary Information [file srep22787-s1.pdf]

Supporting Information

FADD regulates NF-κB activation and promotes ubiquitination of $cFLIP_L$ to induce apoptosis

Kishu Ranjan and Chandramani Pathak*

Department of Cell Biology, School of Biological Sciences and Biotechnology, Indian Institute of Advanced Research, Koba Institutional Area, Gandhinagar-382007, Gujarat, India.

*Corresponding authors: Chandramani Pathak, Department of Cell Biology, School of Biological Sciences and Biotechnology, Indian Institute of Advanced Research, Koba Institutional Area, Gandhinagar-382007, Gujarat, India. Tel: +91-79-60514244, Fax: +91-79-30514110

E-mail: cmpathak@iiar.res.in

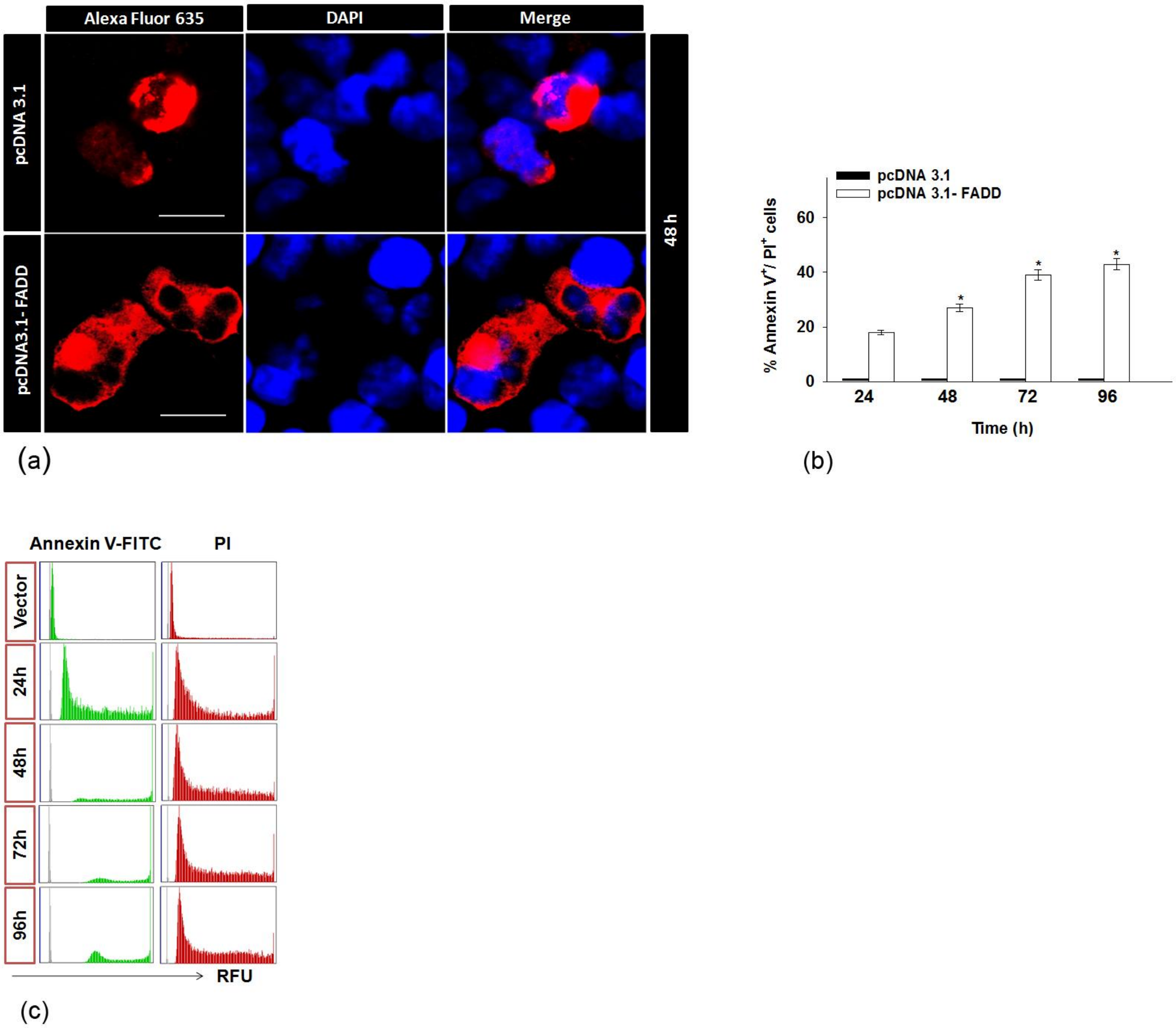

Figure S1. Expression of FADD in HEK 293T cells (a) Immunostaining of FADD in vector transfected cells (endogenous) and pcDNA-FADD transfected cells, post 48 h of incubation, scale bar- 10µm. The pcDNA-FADD was overexpressed for 24-96 h and (b) Percent apoptotic cell death (Annexin-V^+/PI^+) (c) and Annexin-V /PI staining were analyzed by Tali cytometer, control represents vector transfected cells. Error bars represent mean±SD, *P ≤0.05, n ≥ 3, where n is number of independent experiment.

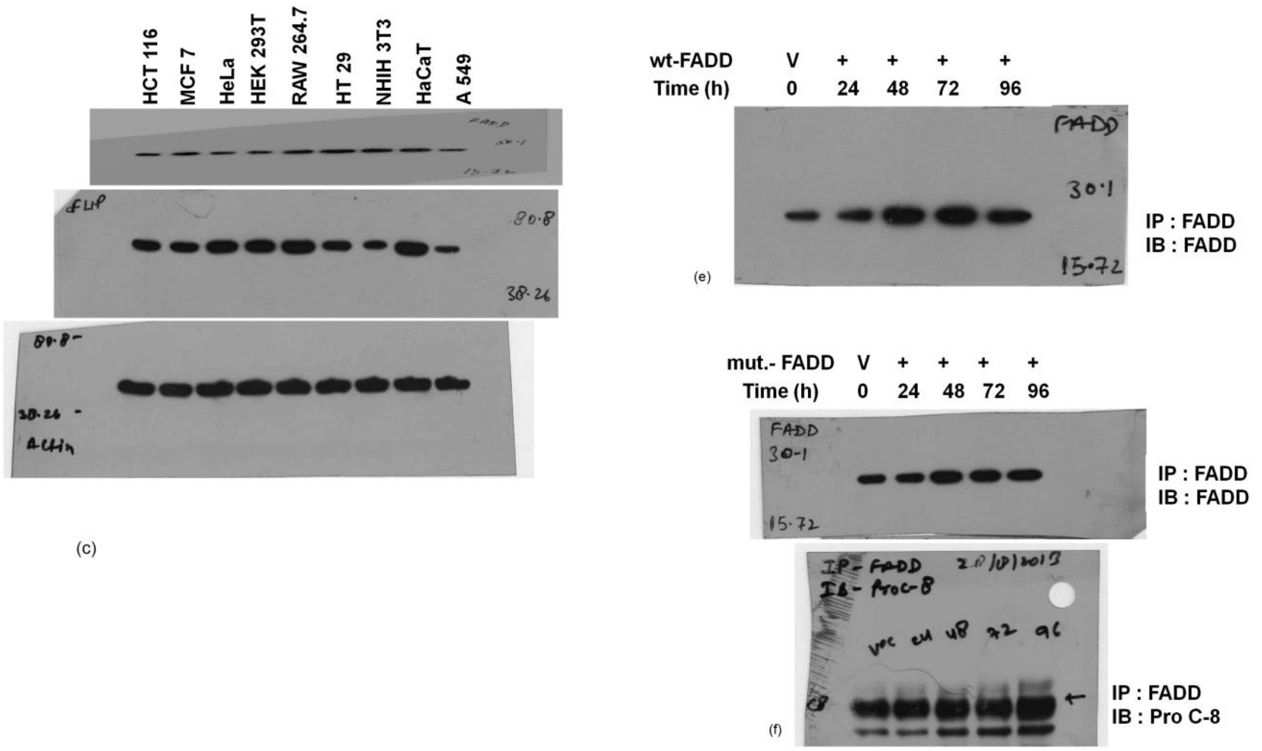

Figure S2. The uncropped full-length image of western blot results for Figure 1c, e & f. The chemiluminescent signals from the exposed membrane were recorded on X-ray films.

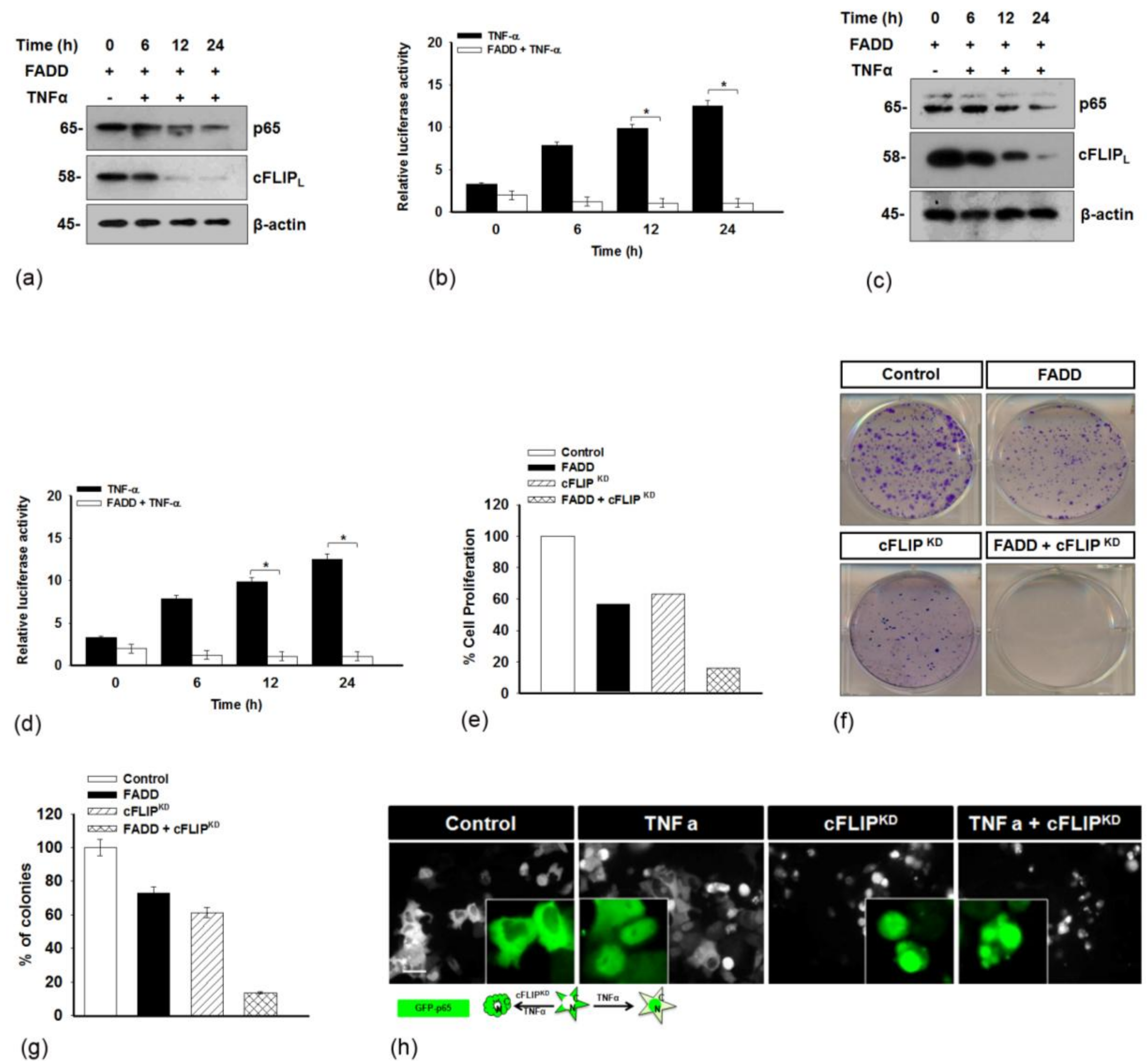

Figure S3. FADD regulates pro-survival response of TNF-α stimulation. TNF-α (10 ng/ml) stimulation to MCF-7 and 48 h of pcDNA-FADD expressed MCF-7 cells to monitor the (a) Expression of p65 and $cFLIP_L$ and (b) NF-κB luciferase reporter activity, TNF-α untreated MCF-7 cells and 48 h of pcDNA-FADD expressed MCF-7 cells (shown as 0 h time point) were taken as control.. TNF-α (10 ng/ml) stimulation to HCT 116 and 48 h of pcDNA-FADD expressed HCT 116 cells to monitor the (c) Expression of p65 and $cFLIP_L$ and (d) NF-κB luciferase reporter activity, TNF-α untreated HCT 116 cells and 48 h of pcDNA-FADD expressed HCT 116 cells (shown as 0 h time point) were taken as control. The uncropped full-length blots are

presented in supplementary Figure S4. HEK 293T cells were transfected with; pcDNA-FADD; (column 2), siRNA directed against $cFLIP_L$ ($cFLIP_L^{KD}$; column 3) and pcDNA-FADD together with siRNA-$cFLIP_L$ (FADD + $cFLIP_L^{KD}$; column 4). Post 48 h of incubation cells were subjected to monitor (e) Percent cell proliferation, (f) Colony forming assay, (g) Percent crystal violet stained colonies and (h) p65 translocation assay, scale bar- 5μm. The vector and non targeting siRNA transfected cells were taken as a control. Error bars represent mean±SD, *P ≤0.05, n ≥ 3, where n is number of independent experiment.

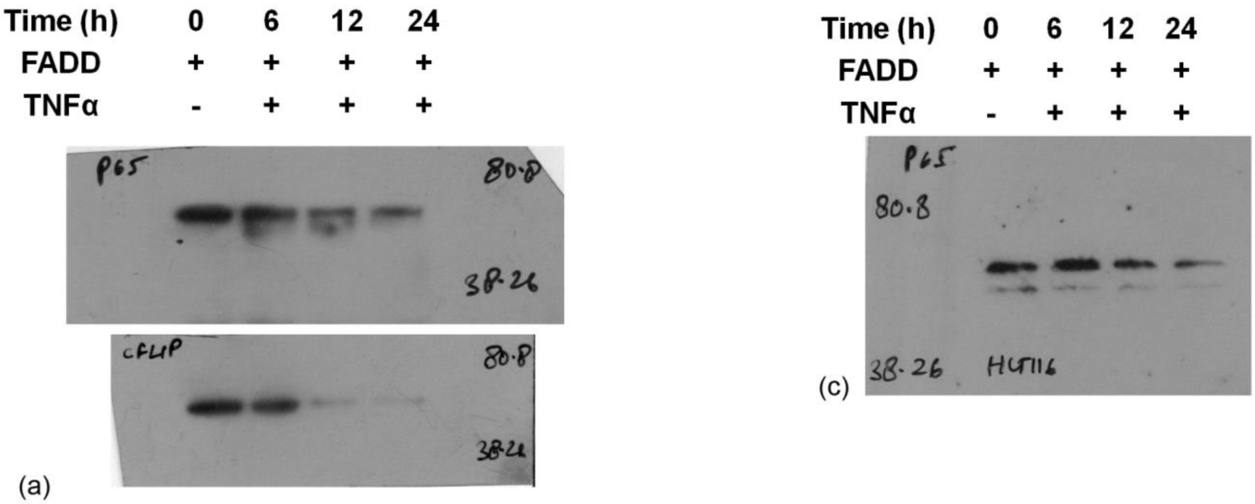

Figure S4. The uncropped full-length image of western blot results for Figure S3a & c. The chemiluminescent signals from the exposed membrane were recorded on X-ray films.

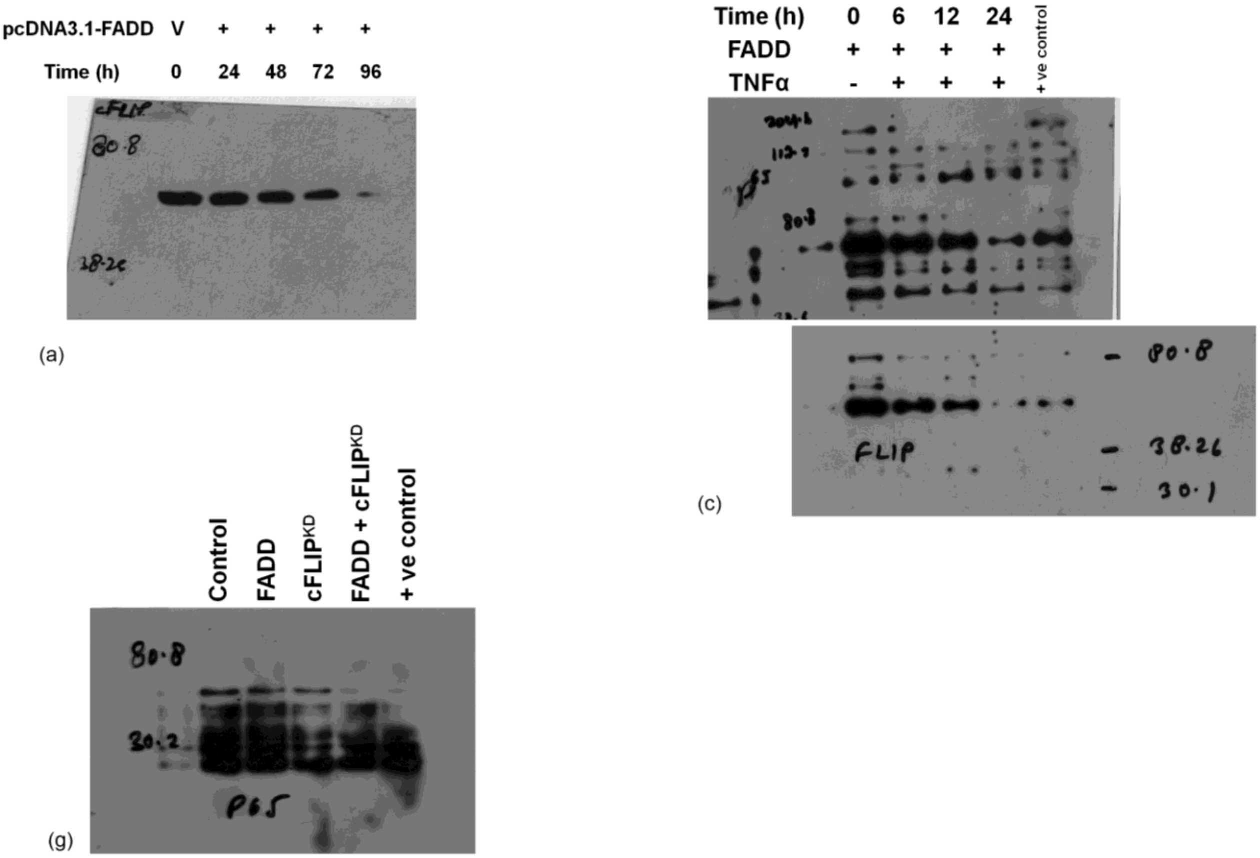

Figure S5. The uncropped full-length image of western blot results for Figure 2a, c & g. The positive control represents IκBα (inhibitor of NF-κB) transfected HEK 293T cells, post 48 h of incubation. The chemiluminescent signals from the exposed membrane were recorded on X-ray films.

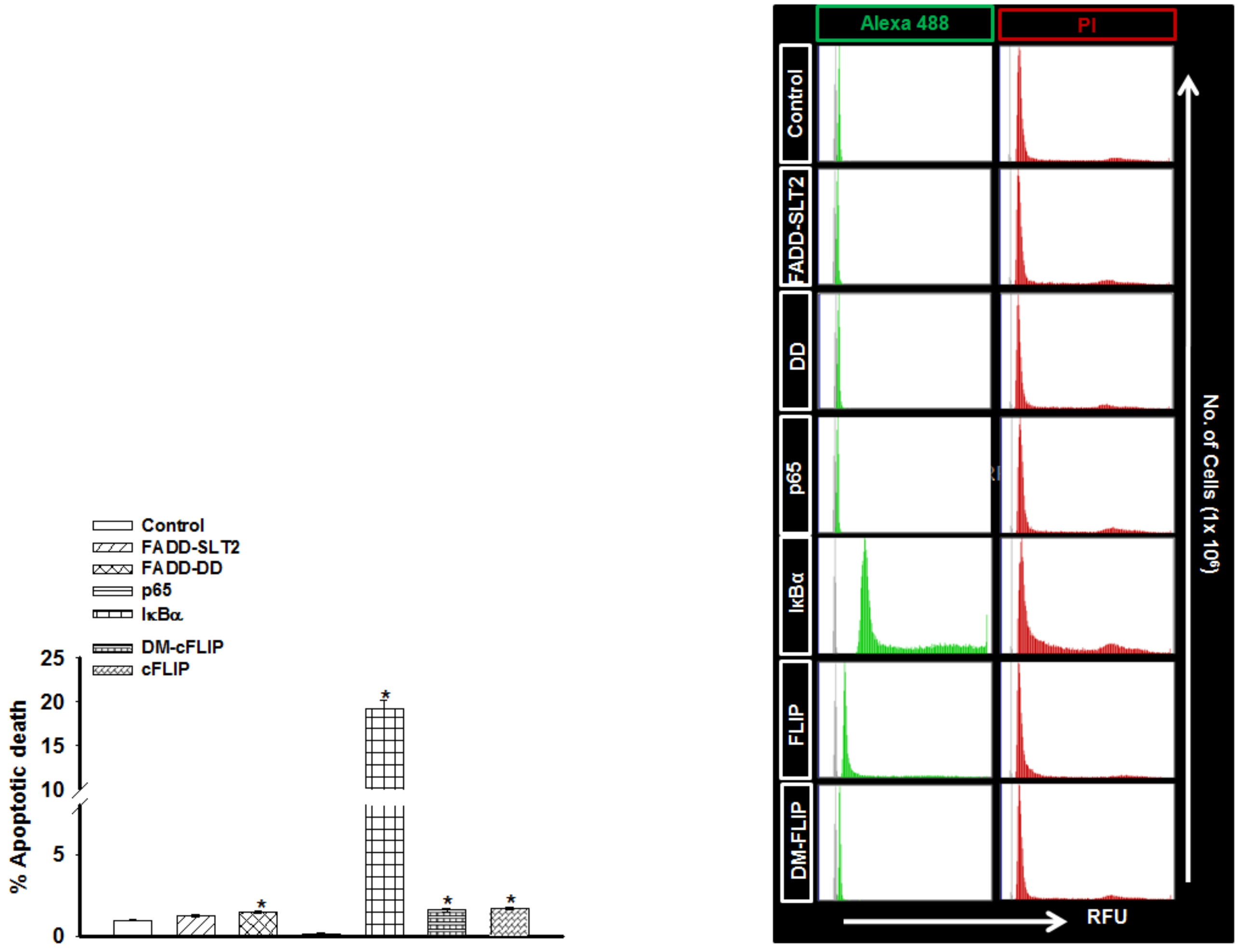

(a) (b)

Figure S6. Effects of domains specific mutation in FADD and cFLIP$_L$ on cell death. The plasmid constructs of wild type (wt) FADD, mutated FADD (FADD-SLT2; **S14G, S16A, S18G, L20A and T21V**), death domain (DD) of FADD (FADD without DED), wt cFLIP$_L$, mutated MC159 (FLIP-DM; an analogous of cFLIP$_L$ unable to activate NF-κB signaling) were transfected to HEK 293T cells for 48 h and (a) Percent apoptotic death and, (b) Annexin-V-alexa 488/PI staining, detected by Tali cytometer, Error bars represent mean ± SD, *P ≤0.05, n ≥ 3, where n is number of independent experiment.

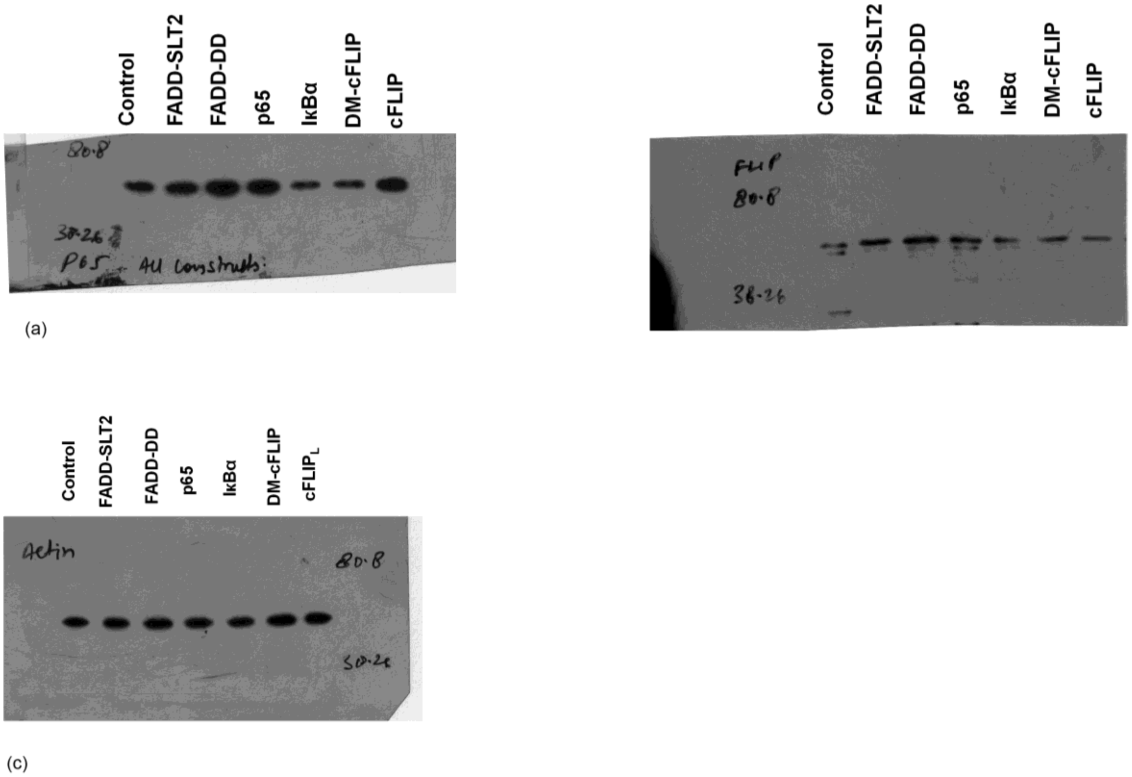

Figure S7. The uncropped full-length image of western blot results for Figure 3c. The chemiluminescent signals from the exposed membrane were recorded on X-ray films.

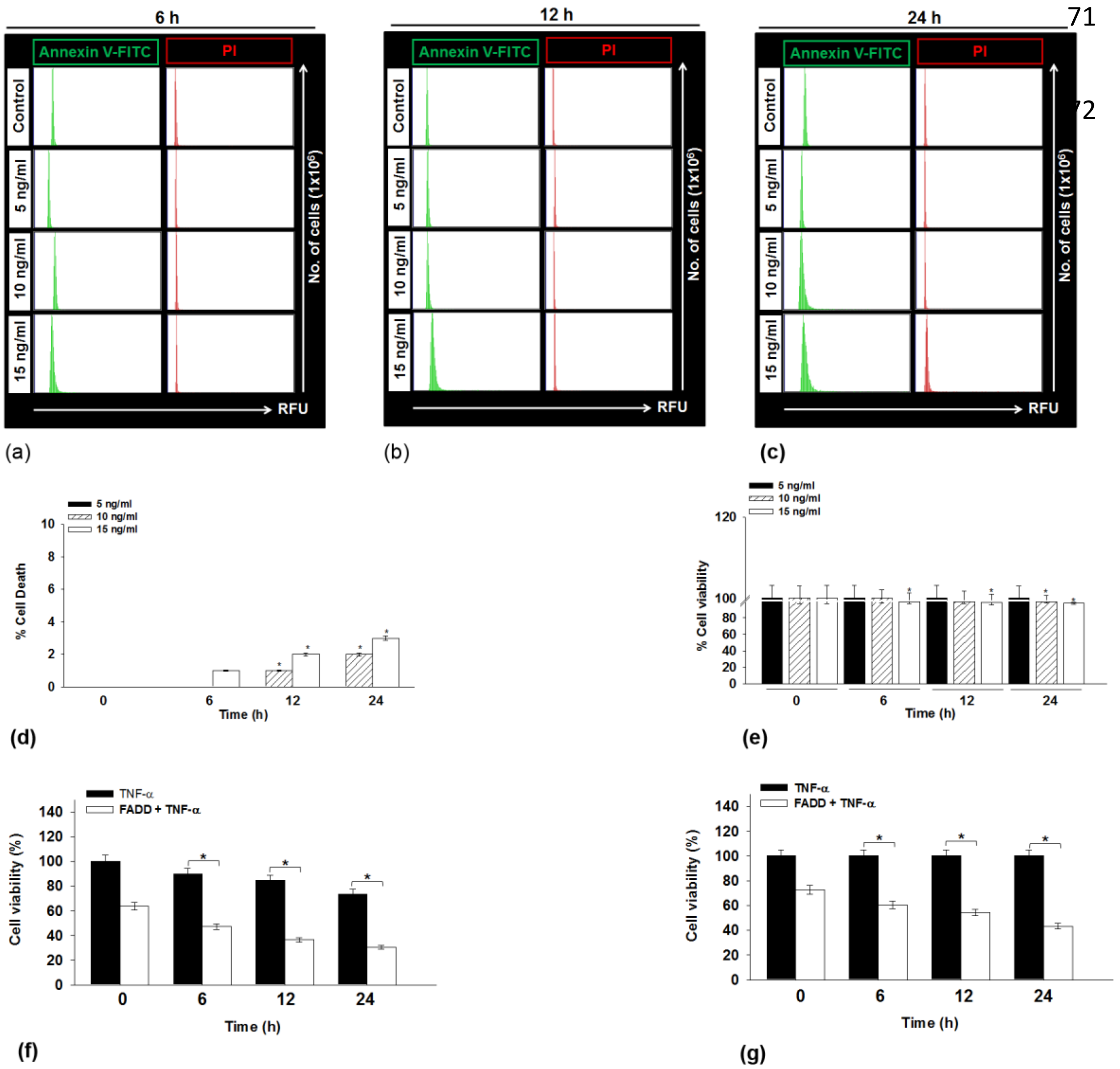

Figure S8. Over expression of FADD commences cell death, independent of TNF-α stimulation. HEK 293T cells were treated with TNF-α (5, 10 and 15 ng/ml) for 6-24 h. Cell death was examined by Annexin V /PI staining detection by automated cell counter Tali™ (a) 6 h, (b) 12 h, (c) 24 h, (d) Percent cell death by by automated cell counter Tali™ and (f) TNF-α (10 ng/ml) stimulation to 48 h of pcDNA-FADD transfected cells to monitor the cell viability in (a) MCF-7, (g) HCT 116 cells and compared with TNF-α treated non-transfected cells. TNF-α untreated and 48 h of FADD expressed cells (shown as 0 h time) were taken as controls. Error bars represent mean±SD, *P ≤0.05, n ≥ 3, where n is number of independent experiment.

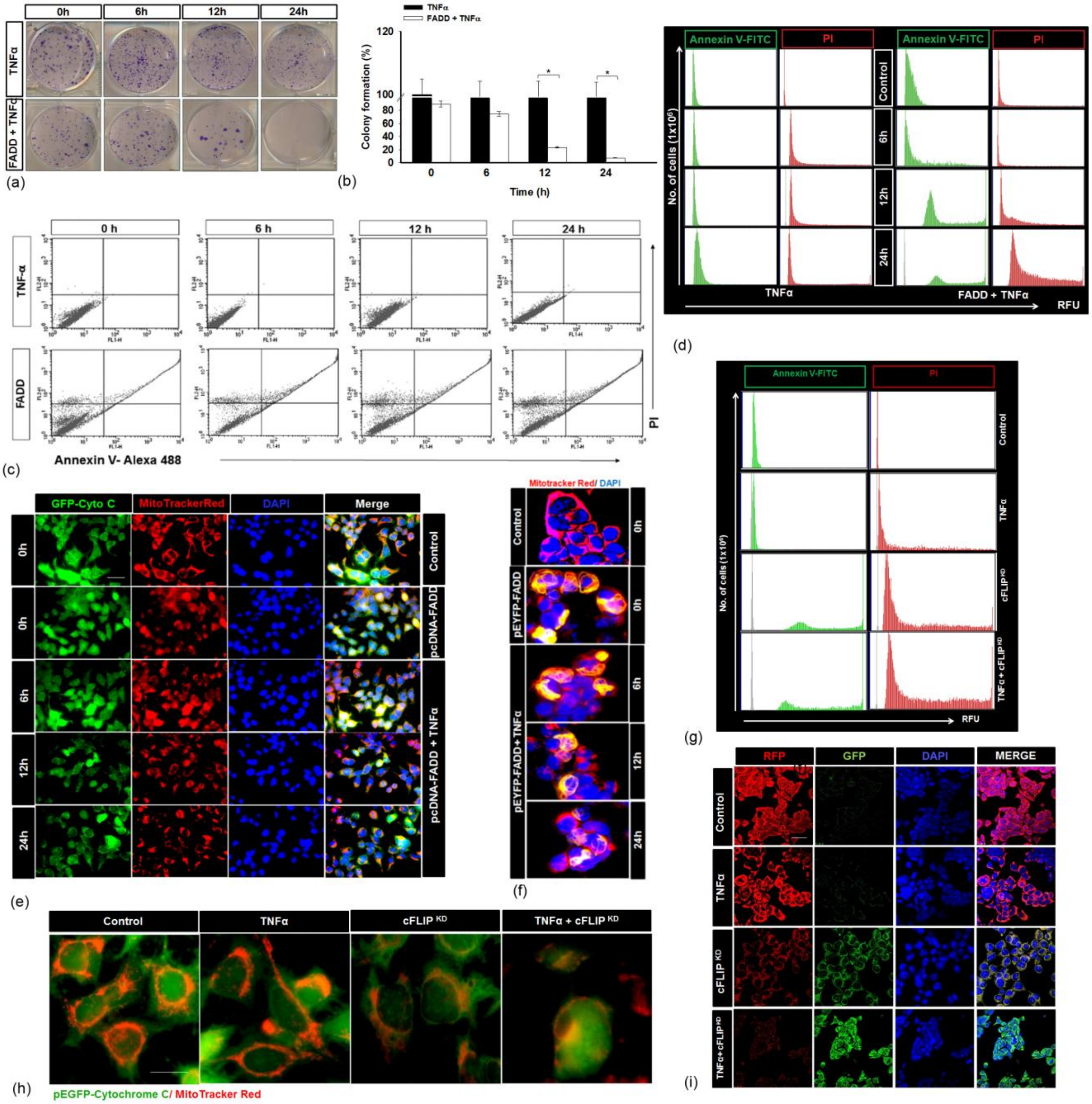

Figure S9. Ablation of NF-κB activation and cFLIP$_L$ expression commences apoptotic cell death. TNF-α (10 ng/ml) stimulation to HEK 293T cells and 48 h of pcDNA-FADD expressed HEK 293T cells. (a) Colony forming assay, (b) Percent crystal violet stained colonies (c) FACS analysis of individually TNF-α treated and FADD expressed cells by BD FACSCalibur™, (d)

Annexin-V-FITC/PI staining detection by Tali cytometer, (e) The pEGFP-cytochrome c stable HEK 293T cells were transfected with pcDNA-FADD (48 h) and further stimulated with TNF-α (10 ng/ml) (f) HEK 293T cells were transfected with pEYFP-FADD (48 h) and treated with TNF-α (10 ng/ml), post incubation cells were stained with MitoTracker Red and counterstained with DAPI, the cells were analyzed under fluorescent microscope, control represents vector transfected cells (shown as 0 min time), scale bar- 5µm. HEK 293T cells were treated with, TNF-α (10 ng/ml) for 12 h (*lane 2*), siRNA-$cFLIP_L$ ($cFLIP_L^{KD}$; *lane 3*) for 48 h and TNF-α (10 ng/ml) primed (12 h) cells followed by siRNA-$cFLIP_L$ (TNF-α + $cFLIP_L^{KD}$; *lane 4*) transfection for an additional 48 h, post incubation, (g) Annexin-V-FITC/PI staining detection by Tali cytometer (h) Qualitative analysis of mitochondrial integrity on pEGFP-cytochrome c stable cells, (i) JC-1 staining. Error bars represent mean±SD, *P ≤0.05, n ≥ 3, where n is number of independent experiment.

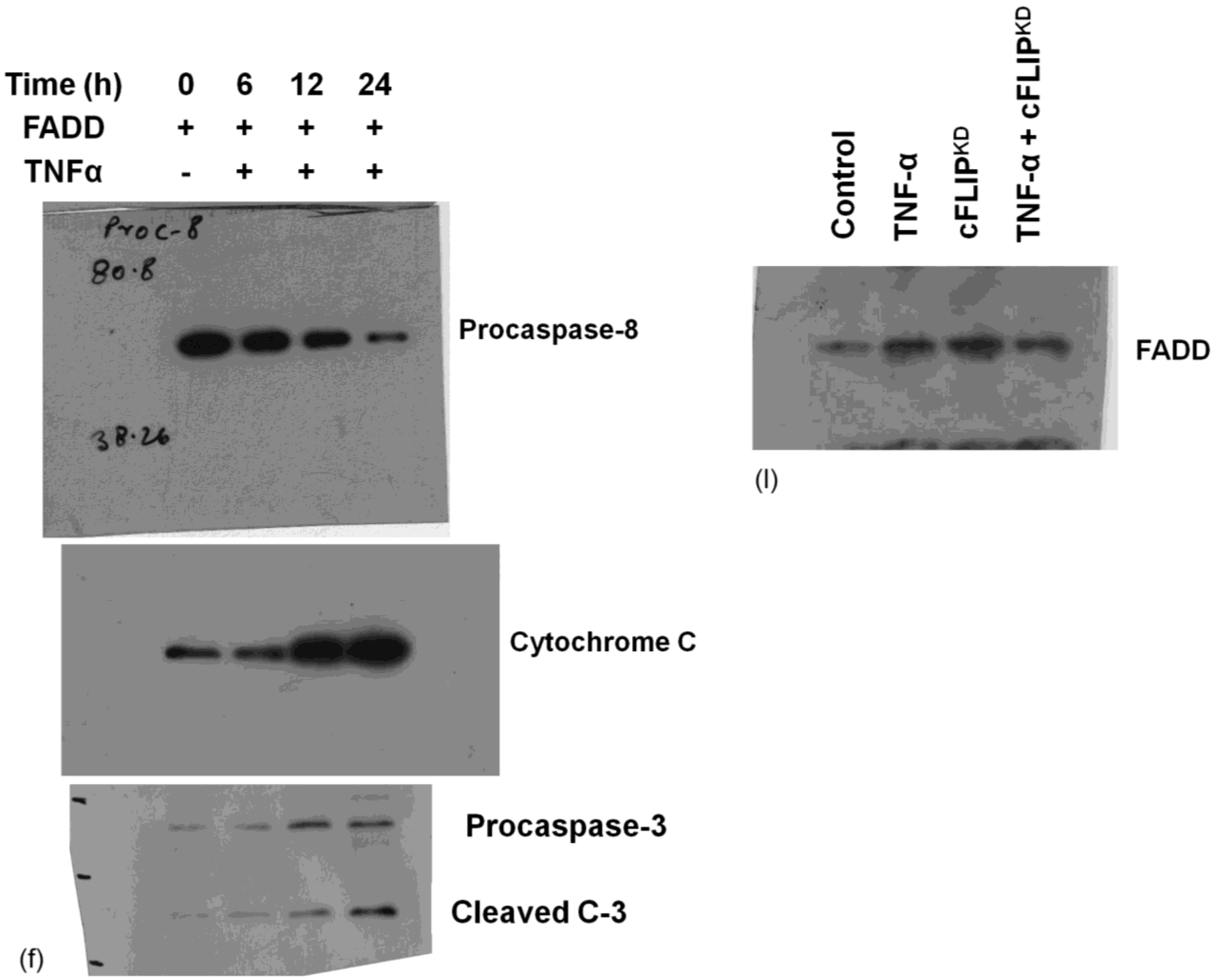

Figure S10. The uncropped full-length image of western blot results for Figure 4f & l. The chemiluminescent signals from the exposed membrane were recorded on X-ray films.

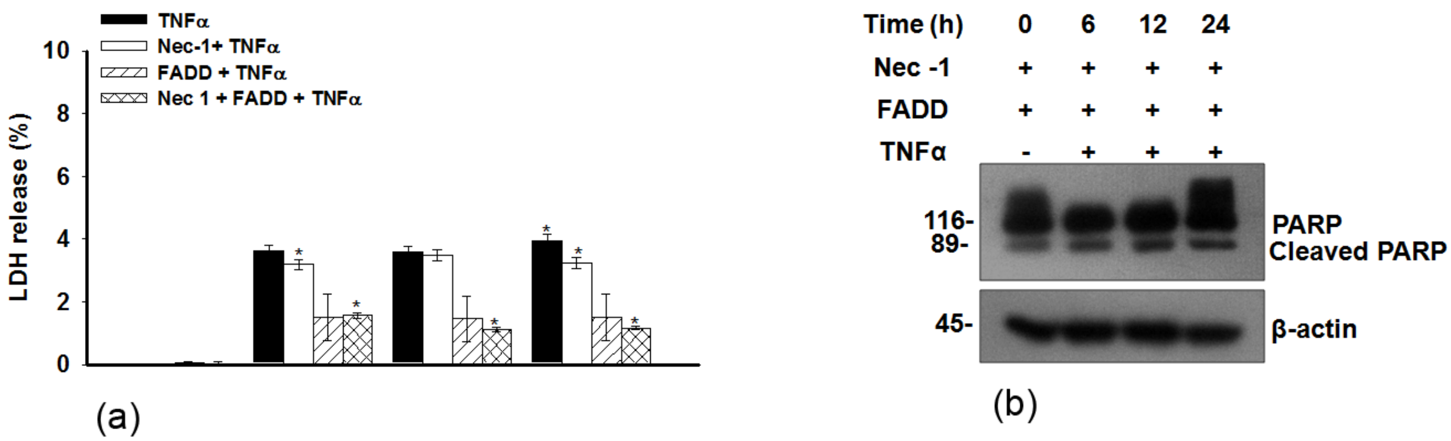

Figure S11. FADD suppresses necrotic death. HEK 293T cells were pretreated with Necrostatin-1 (20μM) for 18 h and further stimulated with TNF-α (10 ng/ml) in HEK 293T and 48 h of pcDNA-FADD expressed HEK 293T cells to monitor (a) Percent LDH release and, (b) Cells were lyzed and the activation of PARP was monitored by western blot analysis. Control represents untreated and FADD expressed cells for 48 h (shown as 0 min time). Error bars represent mean±SD, *P ≤0.05, n ≥ 3, where n is number of independent experiment.

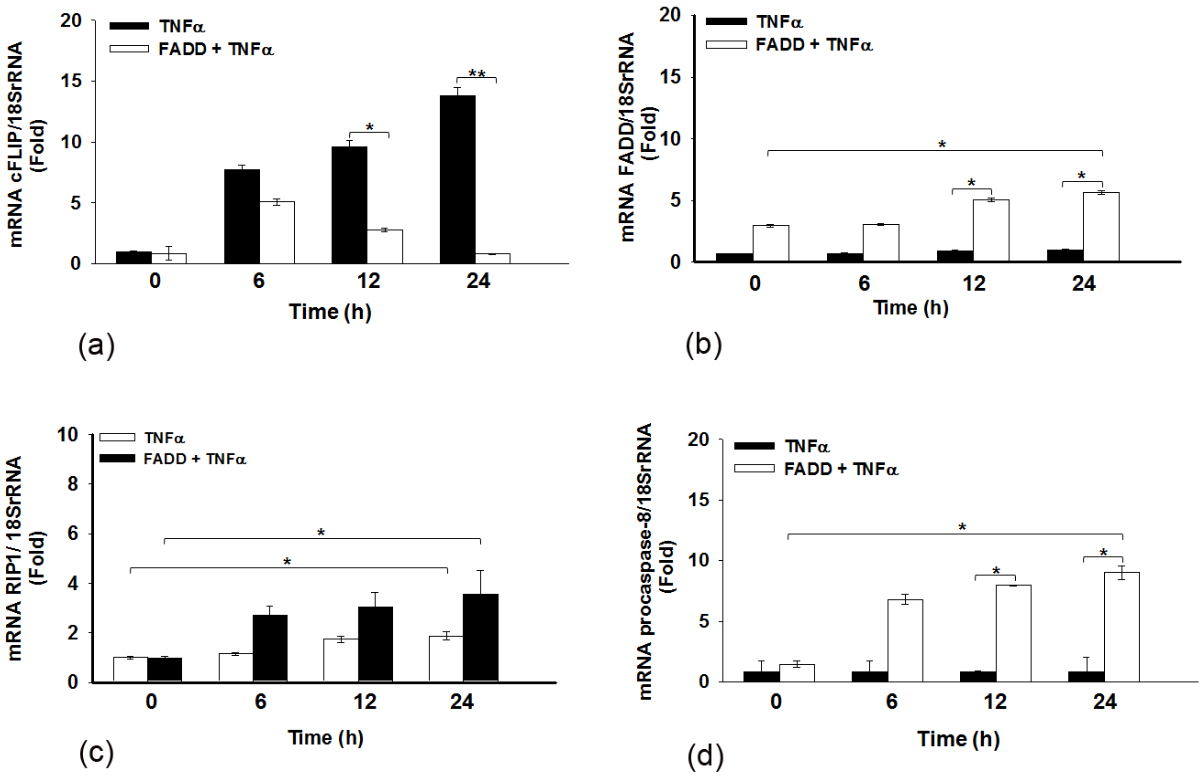

Figure S12. FADD regulates expression of apoptotic components. TNF-α (10 ng/ml) stimulation to HEK 293T and 48 h of pcDNA-FADD expressed HEK 293T cells to analyze the (a) mRNA expression of $cFLIP_L$ (b) mRNA expression of FADD (c) mRNA expression of RIP1 and (d) mRNA expression of Procaspase-8. The values were normalized by using the difference in critical thresholds (C_T) between target gene and 18SrRNA (endogenous control). The expression of mRNA of the respective genes was compared with untreated and 48 h of FADD expressed cells (shown as 0 min time) control using the values of $2^{-\Delta\Delta CT}$. Error bars represent mean±SD, *P ≤0.05, **P ≤0.001, n ≥ 3, where n is number of independent experiment.

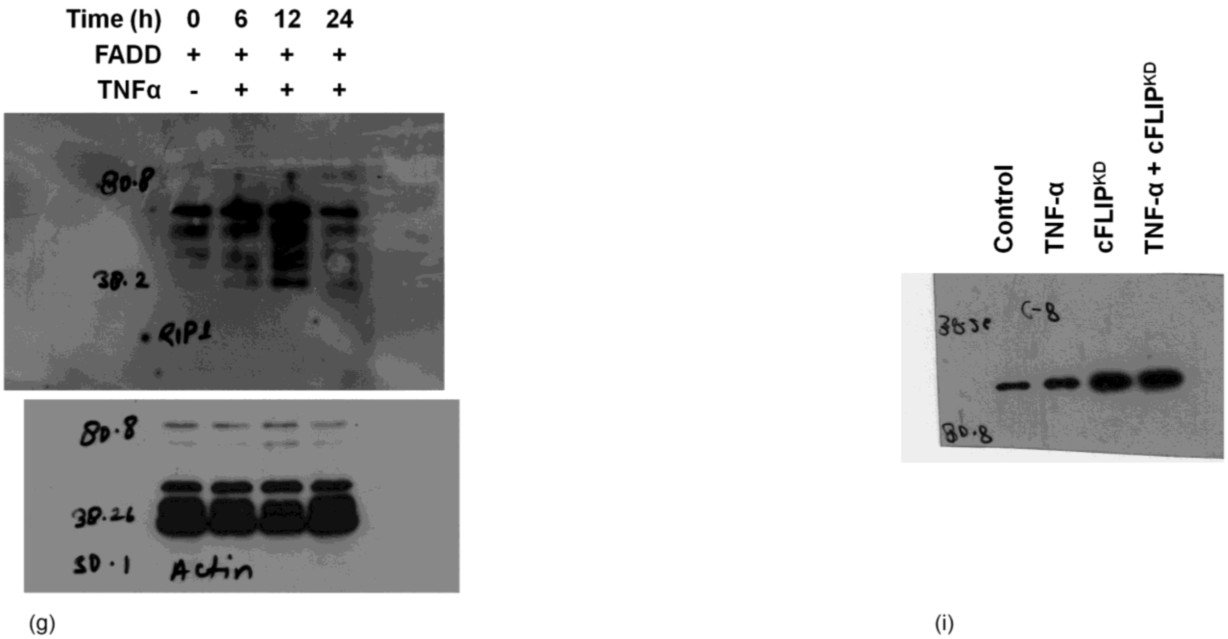

Figure S13. The uncropped full-length image of western blot results for Figure 5g & i. The chemiluminescent signals from the exposed membrane were recorded on X-ray films.

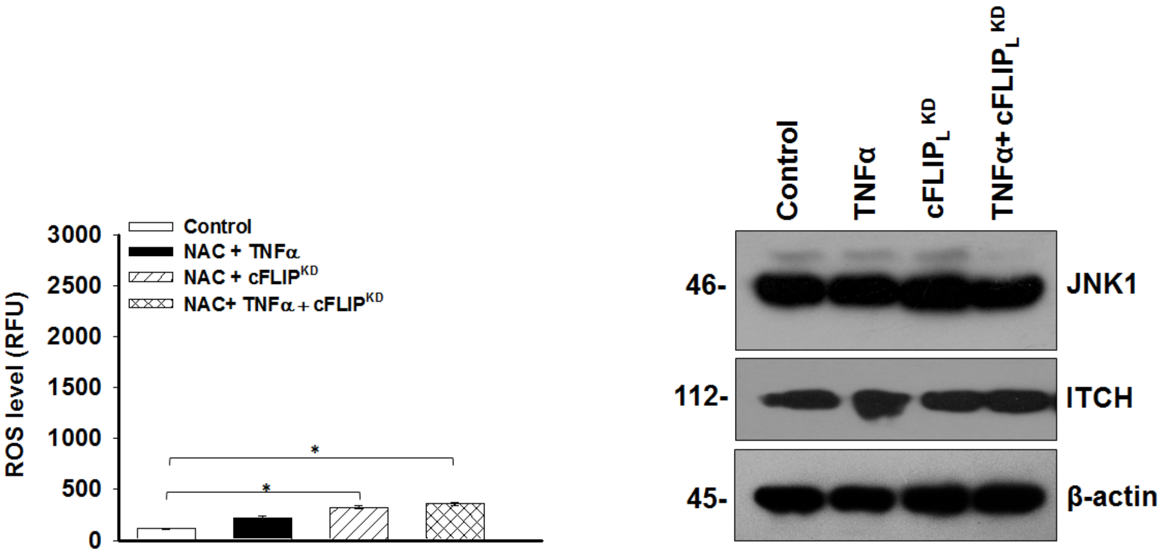

(a) (b)

Figure S14. NAC pretreatment restore ROS mediated JNK activation. HEK 293T cells were pretreated with NAC (25µM) for 3h and further stimulated with TNF-α (10 ng/ml) for 12 h (*bar 2*), transfected with siRNA-cFLIP$_L$ (cFLIP$_L^{KD}$; *bar 3*) for 48 h and TNF-α (10 ng/ml) primed (12 h) cells transfected with siRNA-cFLIP$_L$ (TNF-α + cFLIP$_L^{KD}$; *bar 4*) for an additional 48 h. Post incubation the (a) ROS measurement and (b) Western blot analysis of JNK1 and ITCH. The uncropped full-length blot of JNK1 is presented in supplementary Figure S15. Error bars represent mean±SD, *P ≤0.05, n ≥ 3, where n is number of independent experiment.

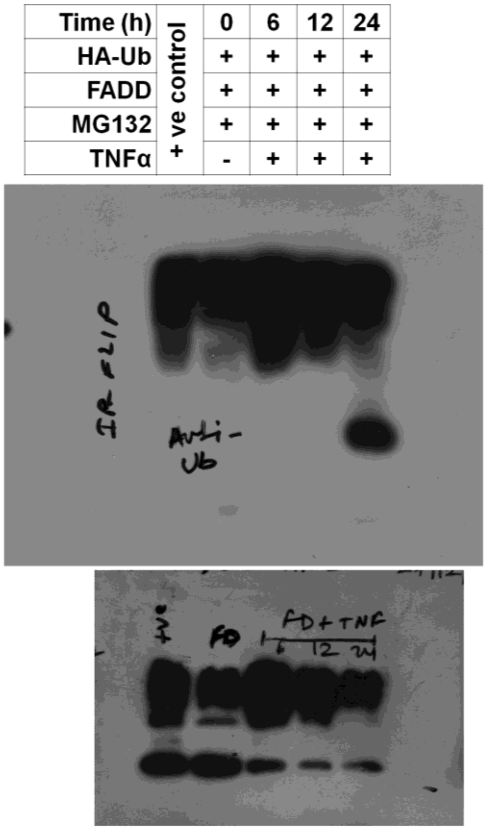

(h)

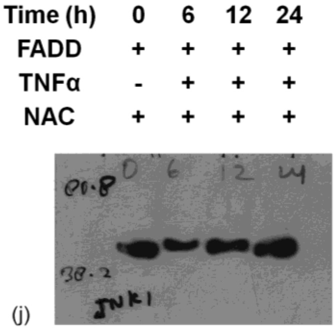

(j)

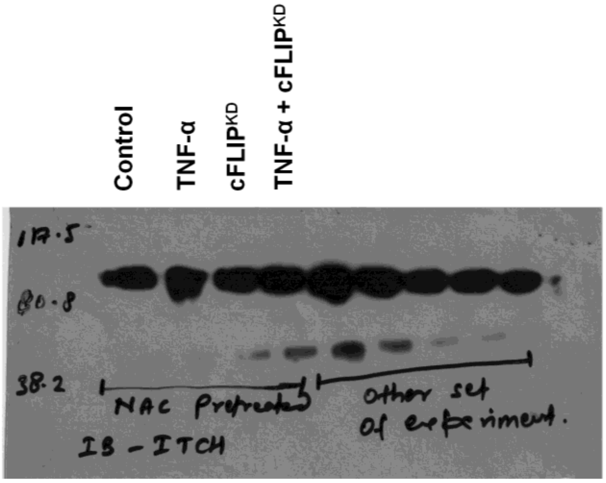

(Figure S13b)

Figure S15. The uncropped full-length image of western blot results for Figure 6h & j and supplementary fig S13b. The positive control represents cycloheximide (5µg/ml for 8 h) treated HEK 293T cells. The chemiluminescent signals from the exposed membrane were recorded on X-ray films.
